# Definitions and surveillance methods of running‐related injuries: A scoping review

**DOI:** 10.1002/ejsc.12123

**Published:** 2024-05-14

**Authors:** Aisling Lacey, Enda Whyte, Sarah Dillon, Siobhán O’Connor, Aoife Burke, Kieran Moran

**Affiliations:** ^1^ School of Health and Human Performance Dublin City University Dublin Ireland; ^2^ Insight SFI Research Centre for Data Analytics Dublin Ireland; ^3^ Centre for Injury Prevention and Performance School of Health and Human Performance Dublin City University Dublin Ireland; ^4^ School of Allied Health University of Limerick Limerick Ireland

**Keywords:** definition, running‐related injuries, surveillance methods

## Abstract

Inconsistent and restricted definitions of injury have contributed to limitations in determining injury rates and identifying risk factors for running‐related injuries (RRIs). The aim of this scoping review was to investigate the definitions and surveillance methods of RRIs. A systematic electronic search was performed using PubMed, Scopus, SPORTDiscuss, MEDLINE, and Web of Science databases. Included studies were published in English between January 1980 and June 2023 which investigated RRIs in adult running populations, providing a definition for a general RRI. Results were extracted and collated. 204 articles were included. Three primary criteria were used to define RRIs: physical description, effect on training and medical intervention, while three secondary criteria are also associated with definitions: cause/onset of injury, location, and social consequences. Further descriptors and sub‐descriptors form these criteria. The use of Boolean operators resulted in nine variations in definitions. Inconsistency is evident among definitions of RRIs. Injury definitions seem to be important for two main reasons: firstly, determining accurate injury rates, and secondly, in research examining risk factors. For the latter, definitions seem to be very limited, only capturing severe injuries and failing to recognise the full development process of RRIs, precluding the identification of conclusive risk factors. A potential two‐approach solution is the initial use of a broad definition acting as a gatekeeper for identifying any potential injury, and follow‐up with an extensive surveillance tool to capture the specific consequences of the varying severity of RRIs.

## Highlights

- There is a clear inconsistency among definitions of what constitutes a running‐related injury (RRI), with frequent use of Boolean operators, ranging criteria and descriptors, and varying terminology and thresholds within these criteria and descriptors.
- The appropriateness of injury definitions and surveillance methods must be considered, with a question of whether they are capable of capturing the entire injury development process.
- To better understand RRIs and identify conclusive risk factors, we must use consistent, reliable and accurate methods of defining and capturing them.

## 1 INTRODUCTION

Running‐related injuries (RRIs) are problematic for runners, being highly prevalent (52%) (Dillon et al., 2023), and associated with negative physical (Hespanhol et al., 2017), psychological (Maschke et al., 2022), social (Sleeswijk Visser et al., 2021), and financial (Hespanhol et al., 2017) consequences. While previous injury (Buist et al., 2010; Hulme et al., 2017) and training errors (Damsted et al., 2018) have been suggested to contribute to the onset of RRIs, there is a lack of agreement on which other factors increase the risk of injury (e.g., running technique, lower limb alignment) (Ceyssens et al., 2019; Fredette et al., 2022; Mousavi et al., 2021; Peterson, Hawke, et al., 2022). Multiple methodological factors contribute to this lack of agreement, including overuse of retrospective studies (Willwacher et al., 2022), one‐off laboratory‐based assessments (Kiernan et al., 2018) and small samples (Kluitenberg et al., 2015). However, inconsistency in the definition of injury is perhaps the most problematic, as evidenced by their impact on incidence rates (Kluitenberg et al., 2015, 2016; Yamato, Saragiotto, Hespanhol Junior, et al., 2015). Yamato and colleagues (Yamato, Saragiotto, Hespanhol Junior, et al., 2015) conducted a systematic review of injury definitions to help establish a consensus definition:

running‐related (training or competition) musculoskeletal pain in the lower limbs that causes a restriction on or stoppage of running (distance, speed, duration or training) for at least 7 days or 3 consecutive scheduled training sessions, or that required the runner to consult a physician or other healthcare professional

(Yamato, Saragiotto, & Lopes, 2015, pp.377).

However, 8 years later, despite improvements in research methodologies (e.g., Running Injury Surveillance Centre (RISC [Burke et al., 2023; Dillon et al., 2021, 2023]), and recommendations for standardised injury registration methods (Kluitenberg et al., 2016), there is still disagreement on injury rates and risk factors for injury (Ceyssens et al., 2019; Fredette et al., 2022; Peterson, Hawke, et al., 2022).

This prompts three questions. Are there still inconsistencies in how RRIs are defined? Are RRIs being defined appropriately? Has Yamato's (Yamato, Saragiotto, & Lopes, 2015) consensus definition been adopted? In relation to the first question, inconsistency in definitions may largely relate to the varied criteria commonly used, such as physical complaints, effects on training, the need for medical attention (Yamato, Saragiotto, Hespanhol Junior, et al., 2015) and the inclusion of Boolean operators (i.e., AND/OR) allowing for either their isolated or combined use. With much RRI research published since the original systematic review (Yamato, Saragiotto, Hespanhol Junior, et al., 2015), an updated investigation is warranted. In relation to the second question, with the strict criteria commonly used to define RRIs (Yamato, Saragiotto, Hespanhol Junior, et al., 2015), definitions may not appropriately represent the true overuse and progressive nature of RRIs (Bertelsen et al., 2017), potentially failing to recognise the lower‐level injuries that runners often train through (Lacey et al., 2023; Soligard et al., 2016; Verhagen et al., 2021). Furthermore, the psychological and social consequences of RRIs (Lacey et al., 2023) are not represented in these definitions. In relation to the third question, since the publication of the initial RRI consensus definition (Yamato, Saragiotto, & Lopes, 2015) no review has examined if it has been adopted.

Lastly, it is important to investigate if RRI data is recorded in a way that captures its progressive nature. With the development of injury surveillance tools (such as the Oslo Sports Trauma Research Center [OSTRC] questionnaires [Clarsen et al., 2013, 2014, 2020]), the capture of overuse injuries has improved. However, while adopted for other sports (Clarsen et al., 2020), it is unknown if and how such tools are being implemented in RRI research.

Therefore, the broad aim of this scoping review is to investigate the registration of RRIs in the literature. This was addressed through the primary aim: to investigate how RRIs are defined by examining: (i) the criteria used to define RRIs, and (ii) how the consensus definition has been adopted since publication, and the secondary aim: to investigate the methods of RRI surveillance.

## 2 METHODS

### 2.1 Protocol and registration

A scoping review was deemed appropriate for the current review as our objective was to broadly examine the registration of RRIs in the literature (i.e., definitions and surveillance methods of RRIs) and map the existing literature on this topic (Grimshaw, 2008; Pollock et al., 2023; Tricco, 2017). The Joanna Briggs Institute Evidence Synthesis and the Preferred Reporting Items for Systematic Reviews and Meta‐Analyses scoping review (PRISMA‐ScR) guidelines (Tricco et al., 2018) were followed as they reflect best practices (Pollock et al., 2023) (see Supporting Information S1: Appendix A for the PRISMA‐ScR checklist). The study protocol for this scoping review has not been previously published; however, it has been registered with Open Science Framework (https://doi.org/10.17605/OSF.IO/UG4JW).

### 2.2 Information sources

As the overall aim of this review was to investigate how RRIs are registered in the literature, only research articles published in academic journal were of interest. The search was therefore restricted to sources from academic journals involving human subjects and published in English between January 1980 and June 2023. Reviews, opinion articles, conference proceedings or posters, case studies, commentaries and study protocols were excluded. The search terms used are available in the supplementary material (Supporting Information S1: Appendix B) and were combined using Boolean phrases. Bibliographies were also searched for articles considered for inclusion.

### 2.3 Search strategy

Four authors (Authors 1,2,3 and 6) determined the patient, concept and context of interest to this review, after which a comprehensive search strategy was developed detailing search terms, limits applied, possible sources of information, and the inclusion and exclusion criteria of articles (Supporting Information S1: Appendices B and C). A systematic search was undertaken by two authors (Authors 1 and 3) on 19^th^ June 2023. PubMed, Scopus, SPORTDiscuss, MEDLINE and Web of Science databases were searched for studies defining RRIs.

### 2.4 Study selection

Articles considered for inclusion were analysed in two phases. Firstly, titles and abstracts were reviewed using predetermined selection criteria (undertaken by Author 1). Secondly, the full texts were independently reviewed by two authors (Authors 1 and 3). If the full text could not be obtained, respective authors were contacted with a request to provide the full text. If the RRI definition applied was not clearly identified on review of the full text, the respective authors were contacted and asked to provide clarity on the definition. Any disagreements were resolved via discussion or third‐party mediation (Author 6).

### 2.5 Methodological quality assessment

With there being no valid scale applicable to the various study designs included in the current review, the quality of information sources was assessed using a method similar to that of previous reviews (Lopes et al., 2012; van Gent et al., 2007; Yamato, Saragiotto, Hespanhol Junior, et al., 2015). The criteria used were (i) description of the inclusion or exclusion criteria of participants, (ii) description of the type of runner included, (iii) use of a standardised method of data collection for all participants, (iv) the data collected directly from participants, and (v) definition of injury clearly provided in the article (Supporting Information S1: Appendix D). Methodological quality was calculated by adding the score of the four criteria, which were assigned a score of 1 for ‘yes’ and 0 for ‘no’, with a maximum score of four. In relation to criteria (*v*) and the inclusion/exclusion criteria of articles in the current review, if the definition of injury was not included in the main body of the article, authors were contacted to retrieve the definition of injury that was used. If the corresponding author responded and provided the definition of injury, this article was subsequently included. If the author was unable to provide the definition of injury, or if there was no response from the author(s) after three attempts of correspondence, these articles were subsequently excluded as no definition of injury could be sourced.

### 2.6 Data extraction

The data extracted process was planned a priori. A data extraction form was developed to extract and summarise the relevant information, guided by the aims of the review. This form was tested in a pilot phase in which two authors (Authors 1 and 3) independently reviewed and extracted data from a percentage (10%) of the included studies. Authors compared their data extraction to ensure consistency and assess the appropriateness of the data extraction form. Through an iterative process, this form was updated in order to ensure the diversity of RRI definitions and surveillance methods were captured appropriately. The full data extraction process was performed independently by two authors (Authors 1 and 3). Extracted data included: authors' name, publication year, study design, study length, study aim, sample size, sex, age, type of runner investigated, injury rate reported (incidence and/or prevalence), injury definition (i.e., criteria used), source of definition (i.e., cited research or custom definitions), measure of injury severity (if applicable), method of surveilling RRIs, and types of data captured. In terms of injury rates, incidence is only reported on in the current review as this was the predominant measure of injury rate that was utilised in included articles. Primary criteria for defining RRIs were identified a priori based on previous findings (Yamato, Saragiotto, Hespanhol Junior, et al., 2015). A content analysis approach was conducted to analyse definitions further (Peters et al., 2020), with descriptors and sub‐descriptors being identified. Microsoft Excel (version 16.75, Microsoft Corporation) was used to perform the analyses.

## 3 RESULTS

### 3.1 Overview of findings

**FIGURE 1:**
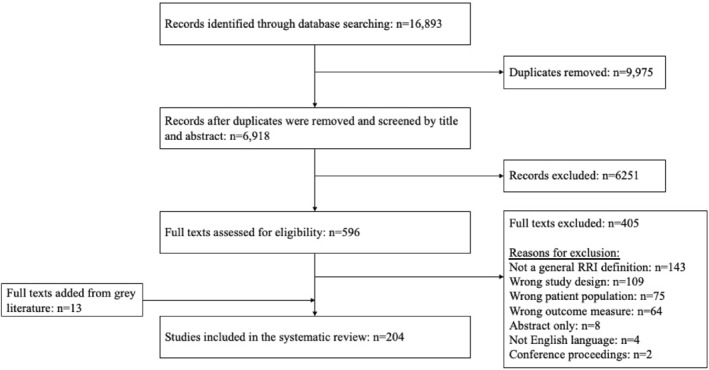
Preferred reporting items for systematic reviews and meta‐analyses scoping review flow diagram. RRI, running‐related injury.

The electronic search identified 16,893 studies. After duplicates were removed (*n* = 9975), 6918 titles and abstracts were screened, and 596 full texts were assessed for eligibility (of which 405 were excluded). Reasons for exclusion were no RRI definition was provided, wrong study design and wrong patient population. Reviewing the bibliographies revealed an additional 13 articles, resulting in 204 articles being included in this review (Figure 1) (Supporting Information S1: Appendix E).

### 3.2 Methodological quality assessment

Sixty percentage (*n* = 123) of studies received a maximum score of five, 32% (*n* = 66) scored four, with the remaining 7% (*n* = 15) receiving a score of three or less. The average score was 4.5. The most common criteria responsible for low quality scores were not providing inclusion or exclusion criteria for participants, and not describing the type of runner included (Supporting Information S1: Appendix E).

### 3.3 Article information

Article publication years ranged from 1981 to 2023, with most studies (80%, *n* = 164) published after 2011 (Supporting Information S1: Appendix F). Among the 204 articles, the majority were prospective (51%, *n* = 105), cross‐sectional (22%, *n* = 44) or retrospective (12%, *n* = 24). Study lengths ranged from 6 days (Bishop & Fallon, 1999) to 12 years (Roberts, 2000). For those that ran longitudinally (*n* = 135), the majority were 6—12 months (28%, *n* = 38), 3—6 months (29%, *n* = 39), or 1 week—3 months (26%, *n* = 35).

### 3.4 Population information

A total of 223,755 participants were included across 204 studies. Seventeen studies did not report sex/gender, and so to those applicable (*n* = 187), 56% (*n* = 80,490) identified as male and 44% (*n* = 60,374) as female. Their average age was 38.2 ± 9.7 years (± refers to standard deviation). One fifth of studies (21%, *n* = 42) did not specify the type of runner; of those that did (*n* = 162), recreational runners were the most common (38%, *n* = 62), followed by novice runners (20%, *n* = 32) and marathon/half‐marathon runners (14%, *n* = 23) (Supporting Information S1: Appendix G).

### 3.5 Criteria used to define RRIs

**FIGURE 2:**
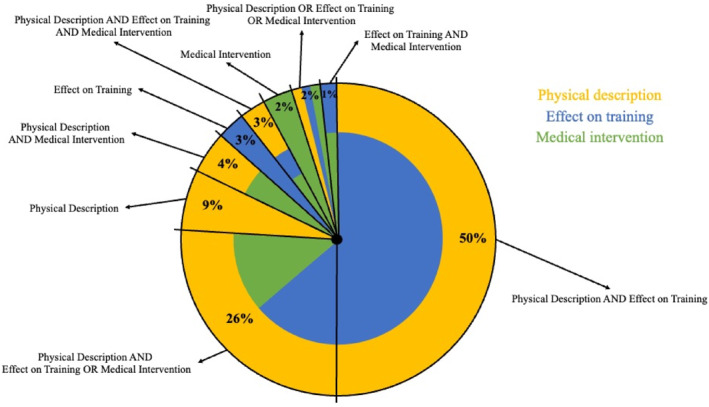
Nine variations of criteria combinations used to define running‐related injuries.

Three primary criteria were used: physical description, effect on training, and requiring medical intervention; and three secondary criteria were used: cause/onset of injury, location of injury, and social consequences. Further descriptors and sub‐descriptors of these criteria are detailed (Supporting Information S1: Appendix H). Overall, ‘physical description’ was the most used criterion (94% of definitions, *n* = 192), followed by ‘effect on training’ (85%, *n* = 174), and requiring ‘medical intervention’ (36%, *n* = 74). Studies engaged with these three criteria either in isolation or in combination (AND/OR), with nine different variations of use (Figure 2); the most frequently used being: (i) physical description AND effect on training (50%, *n* = 103), (ii) physical description AND effect on training OR medical intervention (26%, *n* = 53), and (iii) physical description in isolation (9%, *n* = 19).

#### 3.5.1 Physical description

Where ‘physical description’ was used (94%, *n* = 193), six descriptors were identified: pain, injury, physical complaint, symptom, problem and other. With regard to how these descriptors were used, they were more commonly used in isolation rather than in combination with one another, with pain (in isolation) being the most frequently used (33%, *n* = 63), followed by ‘injury’ (in isolation) (21%, *n* = 40) and physical complaint (in isolation) (16%, *n* = 30) (Supporting Information S1: Appendix I). Just 6% (*n* = 11) of these studies included a minimum time‐frame, ranging from one session to 10 days, with the most common being ‘at least 1 week’ (36%, *n* = 4).

#### 3.5.2 Effect on training

Where ‘effect on training’ was used (85% of total, *n* = 174), two descriptors were identified: ‘time‐loss’ (referring to a complete stop to running), and ‘training restriction’ (referring to a restriction of running) (Supporting Information S1: Appendix J). ‘Training restriction’ in isolation was used in 49% (*n* = 85), ‘time‐loss’ in isolation in 26% (*n* = 45), and ‘time‐loss’ OR ‘training restriction’ in 25% (*n* = 44). For ‘time‐loss’, two descriptors were identified: a missed training session or missed competition. Of all studies that mentioned time‐loss (*n* = 89), the vast majority (96%, *n* = 85) referred to a missed training session in isolation. Additionally, 80% of these (*n* = 71) also mentioned a minimum time‐frame, ranging from ‘at least a partial session’ to ‘at least 4 weeks’. Of these, the majority specified time‐loss of at least 1 week (or three sessions) (55%, *n* = 39), at‐least 1 day/one session (38%, *n* = 27), and more than 1 day but less than 1 week (7%, *n* = 5). In terms of ‘training restriction’, seven sub‐descriptors were commonly used: a general (non‐specific) restriction in training, reduced intensity, reduced duration, reduced frequency, reduced volume, reduced performance and other. Of these (*n* = 128), 27% (*n* = 34) referred to a general (non‐specific) restriction in training, 19% (*n* = 24) referred to a reduction in intensity, duration, frequency OR volume, and 17% (*n* = 22) referred to a reduction in intensity, duration OR volume. Additionally, 63% (*n* = 81) of these studies mentioned a minimum time‐frame, ranging from ‘at least 1 day’ to ‘at least 2 weeks’, with the majority of these (94%, *n* = 76) referring to training restriction of at least 1 week (Supporting Information S1: Appendix K).

#### 3.5.3 Medical intervention

Where ‘medical intervention’ was used (36% of total, *n* = 74), three descriptors were identified: medical attention from a healthcare professional, a need to take medication and specific diagnosis by a healthcare professional (Supporting Information S1: Appendix L). Medical attention alone was the most used descriptor (74%, *n* = 55), followed by medical attention OR taking medication (18%, *n* = 13). Just one study (0.5%) specifically referred to a minimum time‐frame for requiring ‘medical intervention’ (at least one session).

#### 3.5.4 Secondary criteria

Three additional secondary criteria were identified for defining RRIs; however, these were merely used as adjunct criteria: (i) injury cause/onset (75%, *n* = 153), (ii) injury location (72%, *n* = 146), and (iii) social consequences (3%, *n* = 6). Of those that specified injury cause/onset, ‘running‐related’ was the most common descriptor (96%, *n* = 147). Of those that specified injury location, the musculoskeletal system was mentioned most often (84%, *n* = 123), followed by the lower limb (65%, *n* = 95) and the lower back (35%, *n* = 51). Finally, where social consequences were included, negative effects on a runners' daily life (e.g., inability to go to work or school) were mentioned in all (100%, *n* = 6).

#### 3.5.5 Incidence rates

The lowest average incidence rate (5% [Willwacher et al., 2016]) was associated with a definition using ‘physical description’ in isolation, while the highest average (96% [Small & Relph, 2018]) was associated with a ‘physical description’ AND ‘effect on training’ definition (Supporting Information S1: Appendix M). Averaging the incidence rates for each of the nine variations of definition, the lowest average incidence rate was a ‘physical description’ AND an ‘effect on training’ AND ‘medical intervention’ (29% ± 0.1%); however, only two studies employed this definition (Chan et al., 2018; Hendricks & Phillips, 2013). The highest average incidence rate (83%) solely utilised a ‘medical intervention’; however, only one study employed this, and was an ultra‐marathon event (Graham et al., 2021).

There was little difference between the average incidence rates of the most used definitions. A ‘physical description’ AND ‘effect on training’ definition had an average incidence rate of 36% ± 0.2% (*n* = 69), while for ‘physical description’ AND ‘effect on training’ OR ‘medical intervention’ it was 34% ± 0.2% (*n* = 30). The largest difference in average incidence rates appears due to the minimum duration employed. A ‘time‐loss’ or ‘training restriction’ of at‐least 1 day resulted in an average incidence rate of 30%, while for at‐least 1 week it was 43%; a 13% difference.

### 3.6 Adoption of the consensus definition (Yamato, Saragiotto, & Lopes, 2015)

Most definitions used either a single citation or multiple citations (59%, *n* = 121), with 75 different references employed. The remaining 41% (*n* = 83) provide no citation. The most common citation was the consensus definition (Yamato, Saragiotto, & Lopes, 2015), referenced in 19% (*n* = 38) of all studies, followed by Buist et al. (2008) referenced in 6% (*n* = 13). Since the publication of the consensus definition in 2015, its first citation was in 2018 by Besomi et al. (2018). Since then, 108 studies were published, of which 40% (*n* = 44) used the consensus definition either by directly citing it (*n* = 38), or by using the same criteria (*n* = 6) (Supporting Information S1: Appendix N).

Table 1 details how studies used the nine variations of definitions overall, prior to the consensus definition, and after its publication. The use of ‘physical description’ AND ‘effect on training’ remained the most frequently used definition pre‐to post‐consensus publishing. Regarding ‘physical description’ synonyms, use of the term ‘pain’ increased following the consensus definition (36%–49%). With ‘effect on training’, the use of ‘training restriction’ in isolation reduced (47%–36%), while the use of ‘time‐loss’ OR ‘training restriction’ increased (15%–28%). Finally, medical attention was used more frequently (22%–45%).

**TABLE 1:** Comparison of definitions of running‐related injuries pre‐ and post‐consensus definition (Yamato, Saragiotto, & Lopes, 2015).

Definition of injury used	Total studies (*n* = 204)	Pre‐consensus definition (*n* = 95)	Post‐consensus definition (*n* = 109)
Physical AND training	50% (*n* = 103)	62% (*n* = 59)	40% (*n* = 44)
Physical AND training OR medical	26% (*n* = 53)	15% (*n* = 14)	36% (*n* = 39)
Physical	9% (*n* = 19)	8% (*n* = 8)	10% (*n* = 11)
Physical AND medical	4% (*n* = 8)	4% (*n* = 4)	4% (*n* = 4)
Training	3% (*n* = 7)	4% (*n* = 4)	3% (*n* = 3)
Physical AND training AND medical	3% (*n* = 6)	2% (*n* = 2)	4% (*n* = 4)
Medical	2% (*n* = 3)	2% (*n* = 2)	1% (*n* = 1)
Physical OR training OR medical	2% (*n* = 3)	2% (*n* = 2)	1% (*n* = 1)
Training AND medical	1% (*n* = 2)	‐	2% (*n* = 2)

### 3.7 Methods of RRI surveillance

Four methods of injury surveillance were primarily utilised: (i) surveys/questionnaires (84%, *n* = 174), (ii) training diaries (25%,*n* = 52), (iii) interviews/phone calls (5%, *n* = 11), and (iv) medical assessments (5%, *n* = 10). For the surveys/questionnaires (*n* = 174), most sources were not referenced (83%, *n* = 145). Of those that did provide a reference, 20 various references were used, with the most common being the OSTRC (health version [OSTRC‐H] and OSTRC overuse injury version [OSTRC‐O] 5% and 4%, respectively). The OSTRC‐O and OSTRC‐H were first published in 2013 and 2014, respectively. Since their first use by Hespanhol et al. (2016), 13% of the 124 studies used one of the OSTRC tools.

One hundred and sixteen studies captured injury data on repeated occasions, with the majority having captured data weekly (32%, *n* = 37), every session (14%, *n* = 16) or every 2 weeks (13%, *n* = 15) (Appendix O).

## 4 DISCUSSION

Our aim was to investigate the registration of RRIs in the literature, primarily by examining injury definitions in the form of (i) the criteria used to define RRIs, and (ii) how the consensus definition has been adopted since publication, and secondly to investigate the methods of RRI surveillance. The definition of injury is important for two primary reasons. Firstly, inconsistent definitions across studies affect incidence and prevalence rates (Kluitenberg et al., 2015; Yamato, Saragiotto, Hespanhol Junior, et al., 2015; Yamato, Saragiotto, & Lopes, 2015). The use of Boolean phrases, which result in varying definitions, can influence average incidence rates by up to 54%. Additionally, when the minimum time‐frame associated with a definition is considered, a difference of 13% was found between incidence rates. Only one study appears to have directly examined the effect of the definition of injury on the rate of injury (Kluitenberg et al., 2016), similarly finding significant differences in reported injury incidence. Secondly, the definition of injury is important in examining potential risk factors for injury. Should definitions continue to only capture higher‐level injuries and fail to identify complaints (Verhagen et al., 2021) or lower‐level injuries (Lacey et al., 2023), research may fail to identify conclusive risk factors. For example, it is possible that lower‐level injuries may not result in week‐long training restrictions, and would therefore be missed with a definition that requires this length (76% of studies that used a time frame for ‘effect on training’). However, they may lead to alterations in running that may predispose other structures to excessive loading and injury (Wilke et al., 2019), which then do meet the week‐long criterion. If the lower‐level injury is not recorded, its potential role as a risk factor and the associated changes in running technique will be overlooked.

### 4.1 Criteria used to define RRIs

Our findings are similar to a previous review (Yamato, Saragiotto, Hespanhol Junior, et al., 2015) in identifying three primary criteria that are predominately used to define RRIs: physical description, effect on training and requiring medical intervention. Novel to our review, however, is how these criteria are used in isolation and/or in combination, resulting in nine variations of definition (Figure 2), creating inconsistencies. Inconsistency escalates when the sub‐descriptors are additionally considered.

#### 4.1.1 Primary criteria: Physical description

Various synonyms of ‘physical descriptions’ are used to define RRIs, with ‘pain’ the most common (Yamato, Saragiotto, Hespanhol Junior, et al., 2015). This finding aligns with the OSTRC‐O in which respondents are specifically questioned about the extent of ‘pain’ they experience (Clarsen et al., 2013, 2020). However, our findings do not align with the terms used in consensus definitions of injury in other sports, such as athletics (Timpka et al., 2014), soccer (Fuller et al., 2006) or rugby (Fuller et al., 2007). In these definitions, the term ‘physical complaint’ is more generally used, allowing for a broader scope of injury to be captured, and not limited to those injuries only associated with ‘pain’. ‘Pain's’ frequent use, along with its specific inclusion in the consensus definition (Yamato, Saragiotto, & Lopes, 2015), may signify that only ‘adequately severe’ RRIs are being captured. However, RRIs are often associated with a range of alternative physical descriptions, such as tightness, awareness, discomfort or stiffness (Lacey et al., 2023), and if ‘pain’ is continually used in definitions, injuries not strictly associated with ‘pain’ may be omitted. Furthermore, with the perception of ‘pain’ being subjective and individualised (de Jonge et al., 2020; De Oliveira et al., 2021), definitions that insist on ‘pain’ may fail to capture injuries where some runners do not perceive pain, contributing to the inconsistent capture of RRIs.

There is also no clear specification of *when* or for *how long* a ‘physical description’ should be present in order to define a RRI. This review identified that definitions do not specify whether a ‘physical description’ related to injury should be present during running or outside of running. This may cause issues in injury reporting as pain patterns may not always be consistent with running practice. For example, during the development of tendinopathies, a runner may experience tendon pain at the start of a session, but with continued activity the pain subsides and so they disregard the complaint (Kountouris & Cook, 2007; Rio et al., 2014). Toward the opposite end of the injury spectrum, runners have highlighted the importance of the timing of physical complaints (Lacey et al., 2023), describing that pain outside of running sessions (e.g., during activities of daily living) is indicative of more severe injuries (e.g., a muscle strain). Furthermore, when ‘physical description’ is used to define RRIs, it rarely includes a minimum timeframe, offering no guidance to research participants on how long they *should* be experiencing it until it qualifies as an injury.

There are various nuances to consider regarding different types of ‘physical complaint’ and the associated time effect. Inclusion of an umbrella term such as ‘physical complaint’ may be more appropriate than a limiting term (such as ‘pain’). Additionally, the timing of ‘physical descriptions’ should not be limited to training/competition, as often, experiences of ‘physical complaints’ occur outside of these.

#### 4.1.2 Primary criteria: Effect on training

The definitions of RRIs have solely used two descriptors to define how RRIs' affect running: ‘time‐loss’ and ‘training restriction’, with injuries causing time‐loss typically considered more severe (Kluitenberg et al., 2016). While some definitions do not explicitly state ‘time‐loss’ or ‘training restriction’, terms such as ‘prevent’ or ‘stop’ refer to a ‘time‐loss injury’, while ‘restrict’, ‘reduce’ or ‘change’ refer to a ‘training restriction injury’ (Yamato, Saragiotto, Hespanhol Junior, et al., 2015). The sub‐descriptors often associated with this criterion provide clarity on the components of running that are limited by injury. Typically with time‐loss definitions, this limitation is mainly an effect on training (rather than competition), while for ‘training restriction’ definitions, a general, non‐specific effect on training is predominately utilised. Six aspects of training are typically referred to include reduced volume, intensity, duration, frequency, performance etc. This finding builds on a previous review which reported that definitions largely only referred to limited training, running, or distance (Yamato, Saragiotto, Hespanhol Junior, et al., 2015). Our findings, in comparison to Yamato's review (Yamato, Saragiotto, Hespanhol Junior, et al., 2015) signify a change in how RRIs are being defined, as more specificity is being provided on the components of running that RRIs limit. As a novel finding, our review found that very few definitions included a minimum extent of what these restrictions should be (e.g., a percentage in redacted volume), leaving room for interpretation from research participants, and an assumption from readers that any limitation to training is indicative of injury.

Arguably, beyond the type and extent of limitations to training, the minimum length of these limitations appears more important in defining RRIs (Kluitenberg et al., 2016; Yamato, Saragiotto, Hespanhol Junior, et al., 2015). As reflected by our finding of a 13% difference in incidence rates, a requirement for longer interruptions to training in order to define injury appears to underestimate the impact of injury, while the opposite is suggested with shorter interruptions (Kluitenberg et al., 2016; Yamato, Saragiotto, Hespanhol Junior, et al., 2015). Additionally, interruption length affects reported RRI incidence rates, as shorter interruptions (i.e., one‐day vs. one‐week) result in significantly higher incidence rates (Kluitenberg et al., 2016). Our review found that ‘time‐loss’ or ‘training restriction’ of 1‐week (or three consecutive sessions) was most commonly used (Yamato, Saragiotto, Hespanhol Junior, et al., 2015). However, the appropriateness of this time frame should be considered. Firstly, as RRIs develop gradually, runners will often not interrupt training (Clarsen et al., 2013; Lacey et al., 2023; Linton & Valentin, 2018; Lopes et al., 2011; Verhagen et al., 2021). Secondly, when injuries do cause interruptions, they are often interspersed with attempts to continue training (sometimes with modifications), therefore not causing multiple interrupted days in succession (Bahr, 2009; Clarsen et al., 2013; Lacey et al., 2023). Therefore, with the requirement of longer time frames, definitions are potentially missing injuries.

#### 4.1.3 Primary criteria: Medical intervention

Medical attention is the most frequently used ‘medical intervention’ descriptor when defining RRIs (Yamato, Saragiotto, Hespanhol Junior, et al., 2015). Similar to the possible limitations for ‘physical description’ and ‘effect on training’, the use of ‘medical attention’ may result in definitions only allowing for the capture of more severe injuries. We identified that ‘using medication’ was more frequently referred to than a previous review (Yamato, Saragiotto, Hespanhol Junior, et al., 2015), possibly reflecting a change in how runners manage RRIs. Recent findings show that runners primarily self‐manage injuries for as long as possible, only seeking medical attention (from healthcare professionals) when their self‐management has failed, or their injury becomes too severe (Lacey et al., 2023; Peterson, Searle, et al., 2022; Russell & Wiese‐Bjornstal, 2015; Verhagen et al., 2021). By including self‐management strategies (such as ‘use of medication’) in definitions of injury, they may be more likely to capture the complaints (Verhagen et al., 2021) or lower‐level injuries (Lacey et al., 2023) with which runners may not seek medical attention. The appropriateness of specifying ‘medical attention’ in definitions should also be considered in light of associated barriers, such as cost (Hespanhol et al., 2017), the use of self‐management strategies (Lacey et al., 2023; Peterson, Searle, et al., 2022; Russell & Wiese‐Bjornstal, 2015; Verhagen et al., 2021), and a perception that their injuries are not severe enough to warrant medical attention (Grønhaug & Saeterbakken, 2019). By requiring injuries to result in medical attention from healthcare professionals, definitions may also fail to capture those injuries with which runners seek other forms of ‘medical advice’, such as from the Internet or social media (Lacey et al., 2023; Lupton, 2013; Verhagen et al., 2021). Furthermore, a runner may not seek medical attention without first experiencing a ‘physical description’ or an ‘effect on their training’. Therefore, it is unclear if it is necessary to include ‘medical attention’ in definitions.

#### 4.1.4 Secondary criteria

Although the secondary criteria identified in the current review do not directly determine what qualifies an ‘injury’, they provide guidance on the cause/onset (e.g., during running) and the anatomical locations (e.g., musculoskeletal system) of RRIs. The frequent inclusion of the cause/onset and location of injury in definitions is in line with previous research (Yamato, Saragiotto, Hespanhol Junior, et al., 2015). However, as novel findings, our review identifies the scant inclusion of the social consequences of RRIs in definitions, and the absence of consideration of the psychological consequences of injury, despite their occurrence (Hespanhol et al., 2017; Lacey et al., 2023; Russell & Wiese‐Bjornstal, 2015). While not necessary for defining RRIs, methods of data collection should be comprehensive enough to ensure they are being recorded.

### 4.2 Adoption of the RRI consensus definition (Yamato, Saragiotto, & Lopes, 2015)

Following the execution of a similar systematic review, Yamato et al. (Yamato, Saragiotto, Hespanhol Junior, et al., 2015) stated that there was no consistency in the definition of RRIs, inspiring the publication of the consensus definition (Yamato, Saragiotto, & Lopes, 2015) (see Introduction for quote). Our findings reveal that the most used definition includes ‘physical description’ and ‘effect on training’ as criteria, while the second most commonly used definition also mentions ‘medical intervention’. These criteria closely reflect the consensus definition (Yamato, Saragiotto, & Lopes, 2015). Additionally, almost one‐fifth of reviewed studies directly used the consensus definition since its publication, indicating that it is being somewhat adopted. The impact of the consensus definition can perhaps be seen in the following: firstly, the use of the term ‘pain’ is the most common ‘physical description’ synonym; secondly, RRIs are more frequently defined by ‘training restriction’ rather than ‘time‐loss’, a positive reflection on their true consequences; thirdly, ‘medical attention’ remains the most common factor within ‘medical intervention’; finally, at‐least 1 week (or three consecutive sessions) is the most common minimum timeframe for both time‐loss and training restriction. However, inconsistency seems to remain among RRI research as over two fifths of definitions are not accompanied by a citation, implying that these are either custom definitions used by each individual study, or appropriate recognition is not being given to the source of definition used.

### 4.3 Methods of RRI surveillance

No other systematic review has addressed the question of RRI surveillance methods. The team at the OSTRC have reported that their questionnaires have been widely adopted in sports injury research and being utilised by a range of elite sport organisations (e.g., US, Australian and Norwegian Olympic programmes) (Clarsen et al., 2020). Our findings suggest these tools have not been widely adopted in RRI research. While not specifically related to RRIs, the OSTRC questionnaires, in particular the overuse injury version (Clarsen et al., 2013, 2020), are appropriate tools for capturing RRIs as their focus on capturing the signs and symptoms of overuse injuries correlates with the nature of RRI development (Bertelsen et al., 2017), capturing beyond the typically strict criteria and time frames that often define injury. The importance of monitoring ‘lower level injuries’ or non‐time‐loss injuries has been evidenced by research on semi‐professional soccer players (Whalan et al., 2020). With 68% of all time‐loss injuries being preceded by a non‐time‐loss injury (Whalan et al., 2020), these findings highlight the contribution non‐time‐loss injuries have in the development of more serious injuries. Additionally, these findings demonstrate that self‐reporting non‐time‐loss injuries was a ‘good’ predictor of subsequent time‐loss injuries within 7 days (Whalan et al., 2020). It has been suggested that RRI research needs a unified tool that is capable of capturing the entire injury development process (i.e., lower‐level injuries and injuries with significant consequences [i.e., time‐loss or medical attention]) (Kluitenberg et al., 2016), with the running injury continuum potentially being useful in developing such a tool (Lacey et al., 2023).

## 5 RECOMMENDATIONS AND IMPLICATIONS

### 5.1 Recommendations

While we must be mindful to (i) avoid adding to the already evident inconsistencies in injury definitions (Yamato, Saragiotto, Hespanhol Junior, et al., 2015), (ii) understand the positive impact the consensus definition (Yamato, Saragiotto, & Lopes, 2015) has had on RRI research and (iii) build on the recommendations for improvements in injury surveillance (Kluitenberg et al., 2016); our findings allow for several recommendations. Above all, when implementing injury definitions and surveillance methods, researchers should be guided by their research question, design and setting, ensuring that their chosen surveillance strategy allows the aims of their research to be addressed (Nielsen et al., 2020).

In relation to the definition of injury, a question regarding the absoluteness of definitions must be asked considering the findings of the current review. In line with recommendations from the International Olympic Committee for an inclusive definition of injury (International Olympic Committee Injury and Illness Epidemiology Consensus Group et al., 2020), and rather than a runner being considered as strictly injured or uninjured, we suggest that a RRI definition should be inclusive of the entire injury development process, acting as a gatekeeper in identifying the minimum possible level of injury. Due to recent research enhancing our understanding of the breadth of the RRI development process (Lacey et al., 2023; Peterson, Hawke, et al., 2022; Verhagen et al., 2021) and the limitations that definitions can impose in capturing the entirety of this process (as described above), a definition of injury should allow for the recognition of *if or when* a runner is experiencing any level of injury, with further investigation following to explore *which* level of injury they are experiencing. A broader definition of injury will allow for flexibility in investigating all levels of RRIs, as previously, research has shown that non‐time‐loss injuries in soccer increase the risk of time‐loss injuries three‐seven fold (Whalan et al., 2020). Indeed, runners have also clearly described their experiences of lower‐level injuries escalating into more serious time‐loss injuries (Lacey et al., 2023; Verhagen et al., 2021). Therefore, we suggest RRIs be defined by a ‘physical complaint’ definition, not enforcing further criteria such as time‐loss, training restriction, or medical attention requirement:

A musculoskeletal physical complaint of the lower limb or back that results from running, regardless of the extent of consequences sustained.

This definition differs from the consensus definition (Yamato, Saragiotto, & Lopes, 2015) in that the single primary criterion used to define injury is a physical description. A physical description is possibly the baseline criterion of what constitutes an injury, as well as being the most used criterion found in the current review. Use of ‘physical complaint’ will allow for the capture of all physical descriptions which possibly signify physiological changes or tissue damage that may be indicative of injury (Whalan et al., 2020; Wilke et al., 2019), rather than just those severe enough to be associated with pain. Additionally, injury surveillance is suggested to be enhanced by the capture of all physical complaints (Clarsen & Bahr, 2014), therefore the generalisation of this definition allows for any runner who may be experiencing some level of injury to be identified.

This recommendation must be considered in light of possible limitations that have been suggested in relation to the overestimation of injury incidence associated with broad definitions (Yamato, Saragiotto, Hespanhol Junior, et al., 2015), and previous questions posed regarding the accuracy and usefulness of monitoring non‐time‐loss injuries in team sports (Orchard & Hoskins, 2007). There is also difficulty in differentiating between ‘normal responses to training’ that are necessary to provoke positive adaptations to training (if managed appropriately) (Wilke et al., 2019), and those which may signify physiological changes which, if not properly managed, may initiate progression along the injury development process (Clarsen et al., 2013; Lacey et al., 2023; Wilke et al., 2019). This challenge may be ever existent; however, with a ‘physical complaint’ definition, it is less likely that those progressing along the injury development process will be overlooked, and it may be found to be an appropriate biomarker for personal injury prevention systems. Additionally, while we acknowledge the discussion points of the METHODS MATTER meeting during which it was suggested that there is no need for a universally accepted definition of injury (as this will depend on the context and research question) (Nielsen et al., 2020); there have also been numerous consensus statements published over the past 2 decades which provide guidance to researchers in addressing the ongoing challenge of defining and surveilling injuries in sport (Bahr et al., 2018; International Olympic Committee Injury and Illness Epidemiology Consensus Group et al., 2020). This need for consistency in injury surveillance research is evident from these recommendations (International Olympic Committee Injury and Illness Epidemiology Consensus Group et al., 2020), as well as from issues associated with inconsistent definitions (as previously described; [Kluitenberg et al., 2015; Yamato, Saragiotto, Hespanhol Junior, et al., 2015; Yamato, Saragiotto, & Lopes, 2015]).

While the primary purpose of this review has been to examine how RRIs are defined, it may be of value to propose that methods of injury surveillance are more important than the definition of injury. Recent findings suggest that a runner experiencing any level of injury along the running injury continuum may be at risk of injury and should be monitored (both in terms of causative and surveillance research) (Lacey et al., 2023; Whalan et al., 2020). We suggest that a comprehensive tool capable of capturing and monitoring the full scope of RRIs, in line with the running injury continuum (Lacey et al., 2023) (i.e., all levels of injury, all possible consequences, and the nature of the injury), is developed and implemented in RRI research. Research should capture all possible responses to the injury development process (including, but not limited to the physical descriptions, effect on running, management strategies required [medical and non‐medical], social consequences, and psychological responses), while the nature of injury should be detailed through the capture of the cause/onset of injury and the location of injury. Support for capturing beyond the ‘typical’ scope of RRI surveillance and monitoring the biopsychosocial responses to injury is evidenced by runners' explicit description of their experiences of injury (Lacey et al., 2023), and recognition of the importance of incorporating a holistic approach to the management of other musculoskeletal conditions (Laisné et al., 2012). While we have suggested that a broad definition be implemented to identify all of those undergoing the RRI development process, each aspect of injury surveillance should be as specific as possible, providing detailed information to assist future research and clinical practices. This process of a two‐phase approach to injury data collection (i.e., the use of a gatekeeper definition initially and then the use of a broad injury surveillance tool), must be investigated to determine its usefulness. To consider the points made by Nielsen and colleagues (Nielsen et al., 2020), it is possible that, once the entirety of the injury development process and its broad scope of consequences is captured consistently (allowing comparison of findings across research), researchers may be able to apply a definition of injury that specifically addresses the aims of their research post‐hoc. RRI surveillance needs to be expanded to determine if lower‐level injuries lead to more severe injuries, as identified with ‘niggles’ in soccer (Whalan et al., 2020). While this extensive type of injury surveillance may be burdensome for injury recorders (Clarsen et al., 2013), the capabilities of modern technologies to collect, manage and analyse large data (Zadeh et al., 2021) bring researchers potentially closer to understand and prevent RRIs.

### 5.2 Implications

This review has clear implications for researchers investigating both risk factors for RRIs and for injury surveillance. Regarding risk factors for injury, our findings highlight the importance of utilising a broad definition of injury and a comprehensive surveillance tool to ensure that non‐time‐loss injuries (e.g., complaints, lower‐level injuries) are captured and considered as potential risk factors for the development of more serious time‐loss injuries. Capturing and monitoring these lower‐level injuries is possibly the missing link in identifying risk factors for RRIs (Lacey et al., 2023). Regarding injury surveillance research, the use of a broad definition and a comprehensive surveillance tool may more appropriately reflect the full extent of RRI rates, rather than just the ‘tip of the iceberg’ as previously suggested (Clarsen et al., 2013).

In line with previous findings (Kluitenberg et al., 2016), this review highlights that the definition of injury is of high importance for the clinical interpretation of research findings, where clinicians should ensure that both the definition of injury and method of injury surveillance are considered when employing an evidence‐based practice.

## 6 LIMITATIONS

This review should be interpreted considering some limitations. Firstly, we only included studies that provided a general RRI definition, rather than those which defined a specific RRI (e.g., Achilles tendinopathy). Secondly, while we have stated that a definition of injury should ensure the aims of a study can be addressed, due to the extent of included studies in the current review, we were unable to investigate the relationship between definition of injury and study aims. Future research studies should consider exploring this research question. Finally, as it was beyond the scope of this review, injury severity was not examined. As a crucial aspect of injury surveillance research, however (Bahr et al., 2018; Yamato, Saragiotto, Hespanhol Junior, et al., 2015), we suggest future research studies investigate how injury severity has been determined in the literature.

## 7 CONCLUSION

Despite an abundance of RRI research studies (as evidenced by the current review), along with improvements in research methodologies and advancements in technologies used to capture injury, there are large variances in injury incidence rates reported and a lack of consistent evidence supporting conclusive risk factors for RRIs. Clearly, how RRIs have been defined and captured may be the missing link. There is a clear inconsistency among definitions of what constitutes a RRI. While the consensus definition by Yamato (Yamato, Saragiotto, & Lopes, 2015) has helped in providing uniformity and is being somewhat adopted by researchers, inconsistencies remain evident with the frequent use of Boolean operators, the range of criteria and descriptors often included and the varying terminology and thresholds within these criteria and descriptors. A second issue is the appropriateness of definitions and surveillance methods used, with research largely failing to recognise, capture and investigate the full development process of injury. With advancements in our understanding of (i) the extent of the injury development process (Lacey et al., 2023; Peterson, Searle, et al., 2022; Verhagen et al., 2021) and (ii) the vast impact RRIs have on runners (Lacey et al., 2023; Russell & Wiese‐Bjornstal, 2015), the appropriateness of injury definitions and surveillance methods must be considered, with a question of whether they are capable of capturing the entire injury continuum (e.g., from ‘discomfort’ to ‘career‐ending injury’ [Lacey et al., 2023]). As definitions seem to be limited to only capture ‘significant’ injuries, failing to recognise the lower‐level injuries that runners experience is possibly underestimating the true rate of RRIs, ignoring potential risk factors for those significant injuries. Ultimately, to better understand RRIs and identify conclusive risk factors, we must use consistent, reliable and accurate methods of defining and capturing them.

## CONFLICT OF INTEREST STATEMENT

There are no conflicts of interest.

## Supporting information

Supporting Information S1

## Data Availability

All data supporting the findings of this review are available within the paper, its supplementary material and in an online data repository, available at: https://doi.org/10.17605/OSF.IO/CGB2F.
