# Supplementary material for: Definitions and surveillance methods of running‐related injuries: A scoping review
Source: Eur J Sport Sci. 2024 May 14;24(7):950–63. doi: 10.1002/ejsc.12123 (PMC11235823; doi:10.1002/ejsc.12123)
Supplement: Supplementary file 1 — Supporting Information S1 [file EJSC-24-950-s001.docx]

**Definitions and surveillance methods of running-related injuries: A scoping review.**

**Supporting Information.**

Appendix A: Preferred Reporting Items for Systematic Reviews and Meta-Analyses scoping review (PRISMA-ScR) checklist (Tricco et al., 2018).

| **Section** | **Item** | **PRIMSA-ScR checklist item** | **Reported on page #** |
| --- | --- | --- | --- |
| TITLE | | | |
| Title | 1 | Identify the report as a scoping review | 1 |
| ABTRACT | | | |
| Structured summary | 2 | Provide a structured summary that includes (as applicable): background, objectives, eligibility criteria, sources of evidence, charting methods, results, and conclusions that relate to the review questions and objectives. | 1 |
| INTRODUCTION | | | |
| Rationale | 3 | Describe the rationale for the review in the context of what is already known. Explain why the review questions/objectives lend themselves to a scoping review approach. | 2-3 |
| Objectives | 4 | Provide an explicit statement of the questions and objectives being addressed with reference to their key elements (e.g., population or participants, concepts, and context) or other relevant key elements used to conceptualize the review questions and/or objectives. | 3 |
| METHODS | | | |
| Protocol and registration | 5 | Indicate whether a review protocol exists; state if and where it can be accessed (e.g., a Web address); and if available, provide registration information, including the registration number. | 3 |
| Eligibility criteria | 6 | Specify characteristics of the sources of evidence used as eligibility criteria (e.g., years considered, language, and publication status), and provide a rationale. | 3-4 |
| Information sources | 7 | Describe all information sources in the search (e.g., databases with dates of coverage and contact with authors to identify additional sources), as well as the date the most recent search was executed. | 3-4 |
| Search | 8 | Present the full electronic search strategy for at least 1 database, including any limits used, such that it could be repeated. | 4 |
| Selection of sources of evidence | 9 | State the process for selecting sources of evidence (i.e., screening and eligibility) included in the scoping review. | 4 |
| Data charting process | 10 | Describe the methods of charting data from the included sources of evidence (e.g., calibrated forms or forms that have been tested by the team before their use, and whether data charting was done independently or in duplicate) and any processes for obtaining and confirming data from investigators. | 4-5 |
| Data items | 11 | List and define all variables for which data were sought and any assumptions and simplifications made. | 4-5 |
| Critical appraisal of individual sources of evidence | 12 | If done, provide a rationale for conducting a critical appraisal of included sources of evidence; describe the methods used and how this information was used in any data synthesis (if appropriate). | NA |
| Synthesis of results | 13 | Describe the methods of handling and summarizing the data that were charted. | 4-5 |
| RESULTS | | | |
| Selection of sources of evidence | 14 | Give numbers of sources of evidence screened, assessed for eligibility, and included in the review, with reasons for exclusions at each stage, ideally using a flow diagram. | 5 |
| Characteristics of sources of evidence | 15 | For each source of evidence, present characteristics for which data were charted and provide the citations. | 5-6 |
| Critical appraisal within sources of evidence | 16 | If done, present data on critical appraisal of included sources of evidence (see item 12). | NA |
| Results of individual sources of evidence | 17 | For each included source of evidence, present the relevant data that were charted that relate to the review questions and objectives | 5-10 |
| Synthesis of results | 18 | Summarize and/or present the charting results as they relate to the review questions and objectives. | 5-10 |
| DISCUSSION | | | |
| Summary of evidence | 19 | Summarize the main results (including an overview of concepts, themes, and types of evidence available), link to the review questions and objectives, and consider the relevance to key groups. | 10-18 |
| Limitations | 20 | Discuss the limitations of the scoping review process. | 19 |
| Conclusions | 21 | Provide a general interpretation of the results with respect to the review questions and objectives, as well as potential implications and/or next steps. | 19-20 |
| FUNDING | | | |
| Funding | 22 | Describe sources of funding for the included sources of evidence, as well as sources of funding for the scoping review. Describe the role of the funders of the scoping review. | 20 |

Appendix B: Search strategy.

| **Search terms:** | Population: “runner” OR “running” |
| --- | --- |
|  | Outcome: “injur*” |
|  | Variables: “incidence” OR “prevalence” OR “risk” OR “rate” OR “burden” |
| **Limits** | Years: 1980-2023 |
|  | Language: English |
|  | Sample: Humans |
| **Excluded sources** | Review articles |
|  | Case studies |
|  | Commentaries |
|  | Conference proceedings or posters |
|  | Opinion articles |
|  | Study protocols |

Appendix C: Inclusion and exclusion criteria.

| **Inclusion criteria** |
| --- |
| 1. Studies which are prospective or retrospective cohort studies, clinical trials or cross-sectional studies examining running-related injuries. |
| 1. Studies which have investigated adult running populations (novice, recreational, elite, and collegiate athletes). |
| 1. Studies which have investigated lower limb musculoskeletal running-related injuries. |
| 1. Studies which are in English |
| 1. Studies which are fully published research articles. |
| **Exclusion criteria** |
| 1. Not a general running-related injury definition provided:  - Studies which provided a definition for a specific running-related injury (e.g., Achilles tendinopathy), rather than a general running-related injury definition. |
| 1. Wrong study design:  - Review articles, study protocols, or case studies. - Studies which are published as conference proceedings, abstracts, or opinion pieces. |
| 1. Wrong patient population:  - Studies which have investigated cohorts that are not exclusively runners (e.g., track athletes, military personnel) - Studies which have investigated cohorts that are not exclusively adult runners (e.g., child or adolescent runners) |
| 1. No definition of injury available |
| 1. Wrong outcomes:  - Studies which investigated upper limb musculoskeletal running-related injuries. |
| 1. Studies which are not in English |

Appendix D: Description of the criteria used to assess the methodological quality of the information sources

|  | **Criteria** | **Description** |
| --- | --- | --- |
| 1 | Participant eligibility criteria | Studies must have included in the Methods section the inclusion and exclusion criteria for the study participants. Studies that clearly defined the eligibility criteria were given a ‘yes’ answer (1) and studies that did not provide clear eligibility criteria received a ‘no’ answer (0). |
| 2 | Description of the type of runner | Studies that reported the types of runner(s), or described the general characteristics of training, or the type of competition being participated in, allowing the identification of the type of runner(s) being assessed received a ‘yes’ answer (1). Studies that did not describe the type of runner, or where it could not be identified, were given a ‘no’ answer (0). |
| 3 | Standardised method of data collection | If the same methods of data collections were used for all runners, the studies received a ‘yes’ answer (1). If data collection was not standardised for all participants, the study received a ‘no’ answer (0). |
| 4 | Data collected directly from participants | Studies in which the methods of data collected were applied directly to the runner, or where a healthcare professional conducted a medical assessment during the study period, received a ‘yes’ answer (1). Studies where the methods of data collected were applied to someone other than the runner (e.g., trainer), or studies in which a medical assessment was conducted prior to the study period received a ‘no’ answer (0). |
| 5 | Definition of injury provided in the article | If the definition of injury was clearly provided in the main body of the article, the studies received a ‘yes’ answer (1). If the definition of injury was not included in the main body of the article, authors were contacted to retrieve the definition of injury that was used. If the corresponding author responded and provided the definition of injury, this article was subsequently included, but would have received a ‘no’ answer (0). If the author was unable to provide the definition of injury, or if there was no response from the author(s) after three attempts of correspondence, these articles were subsequently excluded as no definition of injury could be sourced. |

Appendix E: List of included articles and methodological quality scores

|  | **Study** | **Methodological quality assessment criteria** | | | | | |
| --- | --- | --- | --- | --- | --- | --- | --- |
|  |  | **1** | **2** | **3** | **4** | **5** | **Total** |
| **1** | (Valliant, 1981) | 1 | 1 | 1 | 1 | 1 | 5 |
| **2** | (Koplan, 1982) | 0 | 1 | 1 | 1 | 1 | 4 |
| **3** | (Hutson, 1984) | 1 | 1 | 1 | 1 | 1 | 5 |
| **4** | (Jacobs & Berson, 1986) | 1 | 1 | 1 | 1 | 1 | 5 |
| **5** | (Blair et al., 1987) | 1 | 1 | 1 | 1 | 1 | 5 |
| **6** | (Bovens et al., 1989) | 1 | 1 | 0 | 1 | 1 | 4 |
| **7** | (Macera, 1989) | 1 | 1 | 1 | 1 | 1 | 5 |
| **8** | (Walter, 1989) | 1 | 1 | 1 | 1 | 1 | 5 |
| **9** | (Fields et al., 1990) | 1 | 0 | 1 | 1 | 1 | 4 |
| **10** | (van Mechelen et al., 1993) | 1 | 1 | 1 | 1 | 1 | 5 |
| **11** | (Jakobsen et al., 1994) | 0 | 1 | 1 | 1 | 1 | 4 |
| **12** | (Van Mechelek et al., 1994) | 1 | 1 | 1 | 1 | 1 | 5 |
| **13** | (Koplan et al., 1995) | 0 | 1 | 0 | 1 | 0 | 2 |
| **14** | (Messier et al., 1995) | 1 | 1 | 1 | 1 | 1 | 5 |
| **15** | (Wen et al., 1997) | 1 | 1 | 1 | 1 | 1 | 5 |
| **16** | (Wen et al., 1998) | 0 | 1 | 0 | 1 | 1 | 3 |
| **17** | (Bishop & Fallon, 1999) | 0 | 1 | 1 | 1 | 1 | 4 |
| **18** | (Duffey et al., 2000) | 1 | 1 | 1 | 1 | 1 | 5 |
| **19** | (Roberts, 2000) | 0 | 1 | 1 | 0 | 1 | 3 |
| **20** | (Chorley et al., 2002) | 0 | 1 | 1 | 1 | 1 | 4 |
| **21** | (Hootman et al., 2002) | 1 | 0 | 1 | 1 | 1 | 4 |
| **22** | (Taunton et al., 2002) | 0 | 0 | 1 | 0 | 1 | 2 |
| **23** | (Taunton et al., 2003) | 0 | 1 | 1 | 1 | 1 | 4 |
| **24** | (Lun, 2004) | 1 | 1 | 1 | 1 | 1 | 5 |
| **25** | (K. E. Gerlach et al., 2005) | 1 | 1 | 1 | 1 | 1 | 5 |
| **26** | (Schache et al., 2005) | 0 | 1 | 1 | 1 | 1 | 4 |
| **27** | (McKean et al., 2006) | 1 | 1 | 0 | 1 | 1 | 4 |
| **28** | (Schwellnus & Stubbs, 2006) | 1 | 0 | 1 | 1 | 1 | 4 |
| **29** | (van Middelkoop et al., 2007) | 1 | 1 | 0 | 1 | 1 | 4 |
| **30** | (Buist et al., 2008) | 1 | 1 | 1 | 1 | 1 | 5 |
| **31** | (K. Gerlach et al., 2008) | 1 | 0 | 1 | 1 | 1 | 4 |
| **32** | (Knobloch et al., 2008)K | 0 | 1 | 1 | 1 | 1 | 4 |
| **33** | (Van Middelkoop et al., 2008a) | 1 | 1 | 0 | 1 | 1 | 4 |
| **34** | (Van Middelkoop et al., 2008b) | 1 | 1 | 0 | 1 | 1 | 4 |
| **35** | (Hesar NG et al., 2009) | 1 | 0 | 1 | 1 | 0 | 3 |
| **36** | (Van Ginckel et al., 2009) | 1 | 1 | 1 | 1 | 1 | 5 |
| **37** | (Buist, Bredeweg, Lemmink, et al., 2010) | 1 | 1 | 1 | 1 | 1 | 5 |
| **38** | (Buist, Bredeweg, Bessem, et al., 2010) | 1 | 1 | 0 | 1 | 1 | 4 |
| **39** | (Hoffman & Fogard, 2011) | 1 | 1 | 0 | 1 | 0 | 3 |
| **40** | (Voight et al., 2011) | 0 | 1 | 1 | 1 | 0 | 3 |
| **41** | (Bredeweg et al., 2012) | 1 | 1 | 1 | 1 | 1 | 5 |
| **42** | (Chang et al., 2012) | 1 | 1 | 1 | 1 | 0 | 4 |
| **43** | (Eskofier et al., 2012) | 0 | 1 | 1 | 1 | 1 | 4 |
| **44** | (Ferreira et al., 2012) | 1 | 1 | 1 | 1 | 1 | 5 |
| **45** | (Hespanhol Junior et al., 2012) | 1 | 1 | 1 | 1 | 1 | 5 |
| **46** | (Vadeboncoeur et al., 2012) | 1 | 1 | 1 | 1 | 0 | 4 |
| **47** | (Bredeweg et al., 2013) | 1 | 1 | 1 | 1 | 1 | 5 |
| **48** | (Ellapen et al., 2013) | 0 | 1 | 1 | 1 | 1 | 4 |
| **49** | (Hamstra-Wright et al., 2013) | 1 | 1 | 1 | 1 | 1 | 5 |
| **50** | (Hendricks & Phillips, 2013) | 1 | 0 | 1 | 1 | 1 | 4 |
| **51** | (L. Hespanhol et al., 2013) | 1 | 1 | 1 | 1 | 1 | 5 |
| **52** | (R. O. Nielsen, Buist, et al., 2013) | 1 | 1 | 1 | 1 | 1 | 5 |
| **53** | (R. O. Nielsen, Cederholm, et al., 2013) | 1 | 1 | 1 | 1 | 1 | 5 |
| **54** | (Ramskov et al., 2013) | 1 | 1 | 1 | 1 | 1 | 5 |
| **55** | (Rasmussen et al., 2013) | 1 | 1 | 1 | 1 | 1 | 5 |
| **56** | (Adriaensens et al., 2014) | 0 | 0 | 1 | 1 | 1 | 3 |
| **57** | (Agresta et al., 2014) | 1 | 1 | 1 | 1 | 1 | 5 |
| **58** | (Hein et al., 2014) | 1 | 1 | 1 | 1 | 1 | 5 |
| **59** | (Hoffman & Krishnan, 2014) | 1 | 1 | 1 | 1 | 0 | 4 |
| **60** | (R. O. Nielsen, Rønnow, et al., 2014) | 0 | 1 | 1 | 1 | 1 | 4 |
| **61** | (R. Ø. Nielsen, Parner, et al., 2014) | 0 | 1 | 1 | 1 | 1 | 4 |
| **62** | (R. O. Nielsen, Buist, et al., 2014) | 1 | 1 | 1 | 1 | 1 | 5 |
| **63** | (R. O. Nielsen, Bertelsen, et al., 2014) | 1 | 1 | 1 | 1 | 1 | 5 |
| **64** | (Ryan et al., 2014) | 1 | 1 | 1 | 1 | 1 | 5 |
| **65** | (Theisen et al., 2014) | 1 | 1 | 1 | 1 | 1 | 5 |
| **66** | (van Poppel et al., 2014) | 0 | 0 | 1 | 1 | 1 | 3 |
| **67** | (Dubois et al., 2015) | 1 | 1 | 1 | 1 | 1 | 5 |
| **68** | (Hotta et al., 2015) | 1 | 1 | 1 | 1 | 1 | 5 |
| **69** | (Kluitenberg et al., 2015) | 1 | 1 | 1 | 1 | 1 | 5 |
| **70** | (Malisoux, Ramesh, et al., 2015) | 1 | 1 | 1 | 1 | 1 | 5 |
| **71** | (Malisoux, Nielsen, et al., 2015) | 1 | 1 | 1 | 1 | 1 | 5 |
| **72** | (Mann et al., 2015) | 1 | 1 | 1 | 1 | 1 | 5 |
| **73** | (Peng et al., 2015) | 1 | 1 | 1 | 1 | 1 | 5 |
| **74** | (RAMSKOV et al., 2015)R | 1 | 1 | 1 | 1 | 1 | 5 |
| **75** | (Switlick et al., 2015) | 1 | 0 | 1 | 1 | 1 | 4 |
| **76** | (I. S. Davis et al., 2016) | 1 | 1 | 1 | 1 | 1 | 5 |
| **77** | (L. Hespanhol, Huisstede, et al., 2016) | 1 | 1 | 1 | 1 | 1 | 5 |
| **78** | (L. Hespanhol, van Mechelen, et al., 2016) | 1 | 0 | 1 | 1 | 1 | 4 |
| **79** | (L. Hespanhol, de Carvalho, et al., 2016) | 1 | 1 | 1 | 1 | 1 | 5 |
| **80** | (Kerr et al., 2016) | 0 | 1 | 1 | 1 | 1 | 4 |
| **81** | (Kluitenberg et al., 2016) | 1 | 1 | 1 | 1 | 1 | 5 |
| **82** | (Malisoux et al., 2016) | 1 | 1 | 1 | 1 | 1 | 5 |
| **83** | (Smits et al., 2016) | 1 | 1 | 1 | 1 | 1 | 5 |
| **84** | (Willwacher et al., 2016) | 1 | 1 | 1 | 1 | 0 | 4 |
| **85** | (van der Worp et al., 2016) | 1 | 1 | 1 | 1 | 1 | 5 |
| **86** | (van Poppel et al., 2016) | 1 | 1 | 1 | 1 | 1 | 5 |
| **87** | (Vernillo et al., 2016) | 1 | 1 | 1 | 1 | 1 | 5 |
| **88** | (Baltich et al., 2017) | 1 | 1 | 1 | 1 | 1 | 5 |
| **89** | (R. B. K. Brund et al., 2017) | 1 | 1 | 1 | 1 | 1 | 5 |
| **90** | (Dudley et al., 2017) | 1 | 1 | 1 | 1 | 1 | 5 |
| **91** | (Fuller et al., 2017) | 1 | 1 | 1 | 1 | 1 | 5 |
| **92** | (L. Hespanhol et al., 2017) | 1 | 1 | 1 | 1 | 0 | 4 |
| **93** | (Paquette et al., 2017) | 1 | 0 | 1 | 1 | 0 | 3 |
| **94** | (Vitez et al., 2017) | 0 | 0 | 1 | 1 | 1 | 3 |
| **95** | (Bertelsen et al., 2018) | 1 | 1 | 1 | 1 | 1 | 5 |
| **96** | (Besomi et al., 2018) | 1 | 1 | 0 | 1 | 0 | 3 |
| **97** | (Chan et al., 2018) | 1 | 1 | 1 | 1 | 1 | 5 |
| **98** | (L. C. Hespanhol et al., 2018) | 1 | 1 | 1 | 1 | 1 | 5 |
| **99** | (Hjerrild et al., 2018) | 1 | 1 | 1 | 1 | 1 | 5 |
| **100** | (Leppe & Besomi, 2018) | 1 | 0 | 1 | 1 | 1 | 4 |
| **101** | (Linton & Valentin, 2018) | 1 | 1 | 1 | 1 | 1 | 5 |
| **102** | (Kemler, Blokland, et al., 2018) | 1 | 1 | 1 | 1 | 1 | 5 |
| **103** | (Kemler, Romeijn, et al., 2018) | 1 | 1 | 1 | 1 | 1 | 5 |
| **104** | (Mulvad et al., 2018) | 1 | 1 | 1 | 1 | 1 | 5 |
| **105** | (Napier et al., 2018) | 1 | 1 | 1 | 1 | 1 | 5 |
| **106** | (Ramskov, Rasmussen, Sørensen, et al., 2018) | 1 | 1 | 1 | 1 | 1 | 5 |
| **107** | (Ramskov, Rasmussen, Sorensen, et al., 2018) | 1 | 1 | 1 | 1 | 1 | 5 |
| **108** | (Small & Relph, 2018) | 0 | 1 | 1 | 1 | 1 | 4 |
| **109** | (Tillander et al., 2018) | 0 | 1 | 1 | 1 | 1 | 4 |
| **110** | (van Poppel et al., 2018) | 1 | 1 | 1 | 1 | 1 | 5 |
| **111** | (Vlahek & Matijević, 2018) | 1 | 0 | 1 | 1 | 1 | 4 |
| **112** | (Begizew et al., 2019) | 1 | 1 | 1 | 1 | 1 | 5 |
| **113** | (Besomi et al., 2019) | 1 | 0 | 1 | 1 | 1 | 4 |
| **114** | (R. Brund et al., 2019) | 1 | 1 | 1 | 1 | 1 | 5 |
| **115** | (Cahanin et al., 2019) | 1 | 1 | 1 | 1 | 1 | 5 |
| **116** | (Dallinga et al., 2019) | 1 | 1 | 1 | 1 | 1 | 5 |
| **117** | (Damsted, Parner, Sørensen, Malisoux, Hulme, et al., 2019) | 1 | 0 | 1 | 1 | 1 | 4 |
| **118** | (Damsted, Parner, Sørensen, Malisoux, & Nielsen, 2019) | 1 | 0 | 1 | 1 | 1 | 4 |
| **119** | (Fokkema, de Vos, van Ochten, et al., 2019) | 1 | 0 | 1 | 1 | 1 | 4 |
| **120** | (Fokkema, de Vos, Bierma-Zeinstra, et al., 2019) | 1 | 1 | 1 | 1 | 1 | 5 |
| **121** | (T. P. C. Franke et al., 2019) | 1 | 1 | 1 | 1 | 1 | 5 |
| **122** | (Melgares et al., 2019) | 1 | 1 | 1 | 1 | 1 | 5 |
| **123** | (Onal et al., 2019) | 0 | 1 | 1 | 1 | 1 | 4 |
| **124** | (Payne et al., 2019) | 0 | 1 | 1 | 1 | 1 | 4 |
| **125** | (Perez-Morcillo et al., 2019) | 1 | 1 | 1 | 1 | 1 | 5 |
| **126** | (Relph & Small, 2019) | 0 | 1 | 1 | 1 | 1 | 4 |
| **127** | (Smits et al., 2019) | 1 | 1 | 1 | 1 | 1 | 5 |
| **128** | (Tenforde et al., 2019) | 0 | 1 | 1 | 1 | 1 | 4 |
| **129** | (Van Oeveren et al., 2019) | 0 | 0 | 1 | 1 | 1 | 3 |
| **130** | (Wiegand et al., 2019) | 1 | 0 | 1 | 1 | 1 | 4 |
| **131** | (Benca et al., 2020) | 0 | 1 | 1 | 1 | 1 | 4 |
| **132** | (Costa et al., 2020) | 1 | 1 | 1 | 1 | 1 | 5 |
| **133** | (Craddock et al., 2020) | 1 | 1 | 1 | 1 | 1 | 5 |
| **134** | (de Jonge et al., 2020) | 0 | 1 | 1 | 1 | 1 | 4 |
| **135** | (Dijkhuis et al., 2020) | 0 | 1 | 1 | 1 | 1 | 4 |
| **136** | (Fokkema et al., 2020) | 1 | 1 | 1 | 1 | 1 | 5 |
| **137** | (Hofstede et al., 2020) | 1 | 1 | 1 | 1 | 1 | 5 |
| **138** | (Jauhiainen et al., 2020) | 1 | 1 | 1 | 1 | 1 | 5 |
| **139** | (Johnson et al., 2020) | 1 | 0 | 1 | 1 | 1 | 4 |
| **140** | (Jungmalm et al., 2020) | 1 | 0 | 1 | 1 | 1 | 4 |
| **141** | (Letafatkar, Rabiei, & Afshari, 2020) | 1 | 1 | 1 | 1 | 1 | 5 |
| **142** | (Letafatkar, Rabiei, Farivar, et al., 2020) | 1 | 0 | 1 | 1 | 1 | 4 |
| **143** | (Malisoux et al., 2020) | 1 | 1 | 1 | 1 | 1 | 5 |
| **144** | (Matos et al., 2020) | 1 | 1 | 1 | 1 | 1 | 5 |
| **145** | (Moreno et al., 2020) | 0 | 1 | 1 | 1 | 1 | 4 |
| **146** | (Taddei et al., 2020) | 1 | 0 | 1 | 1 | 1 | 4 |
| **147** | (Tenforde et al., 2020) | 1 | 0 | 1 | 1 | 1 | 4 |
| **148** | (Torres et al., 2020) | 1 | 1 | 1 | 1 | 1 | 5 |
| **149** | (Veras et al., 2020) | 1 | 1 | 1 | 1 | 1 | 5 |
| **150** | (Winter et al., 2020) | 1 | 1 | 1 | 1 | 1 | 5 |
| **151** | (J. Davis & Gruber, 2021) | 1 | 1 | 1 | 1 | 1 | 5 |
| **152** | (DE OLIVEIRA et al., 2021)D | 1 | 1 | 1 | 1 | 1 | 5 |
| **153** | (G. A. Desai & Gruber, 2021) | 1 | 1 | 1 | 1 | 1 | 5 |
| **154** | (P. Desai et al., 2021) | 1 | 1 | 1 | 1 | 1 | 5 |
| **155** | (Dillon et al., 2021) | 1 | 1 | 1 | 1 | 1 | 5 |
| **156** | (T. Franke et al., 2021) | 1 | 1 | 1 | 1 | 1 | 5 |
| **157** | (Gajardo-Burgos et al., 2021) | 1 | 1 | 1 | 1 | 1 | 5 |
| **158** | (Graham et al., 2021) | 0 | 1 | 1 | 1 | 0 | 3 |
| **159** | (Gruber et al., 2021) | 0 | 1 | 1 | 1 | 1 | 4 |
| **160** | (Gutierrez-Hellin et al., 2021) | 0 | 1 | 1 | 1 | 1 | 4 |
| **161** | (L. Hespanhol et al., 2021) | 1 | 1 | 1 | 1 | 1 | 5 |
| **162** | (Holmes et al., 2021) | 1 | 0 | 1 | 1 | 1 | 4 |
| **163** | (Hollander et al., 2021) | 1 | 1 | 1 | 1 | 1 | 5 |
| **164** | (Kliethermes et al., 2021) | 1 | 1 | 1 | 1 | 1 | 5 |
| **165** | (Koech et al., 2021) | 1 | 1 | 1 | 1 | 1 | 5 |
| **166** | (Luedke & Rauh, 2021) | 1 | 1 | 1 | 1 | 1 | 5 |
| **167** | (Malisoux et al., 2021) | 1 | 1 | 1 | 1 | 1 | 5 |
| **168** | (Mayne et al., 2021) | 1 | 1 | 1 | 1 | 1 | 5 |
| **169** | (Mohseni et al., 2021) | 0 | 1 | 1 | 1 | 0 | 3 |
| **170** | (Mousavi et al., 2021) | 1 | 1 | 1 | 1 | 1 | 5 |
| **171** | (Nakaoka et al., 2021) | 1 | 1 | 1 | 1 | 1 | 5 |
| **172** | (Quirino et al., 2021) | 1 | 1 | 1 | 1 | 1 | 5 |
| **173** | (Rhim et al., 2021) | 1 | 1 | 1 | 1 | 1 | 5 |
| **174** | (Sanfilippo et al., 2021) | 1 | 0 | 1 | 1 | 1 | 4 |
| **175** | (Sleeswijk Visser et al., 2021) | 1 | 1 | 1 | 1 | 1 | 5 |
| **176** | (Warne et al., 2021) | 1 | 0 | 1 | 1 | 1 | 4 |
| **177** | (Willems et al., 2021) | 1 | 0 | 1 | 1 | 1 | 4 |
| **178** | (Viljoen et al., 2021) | 1 | 1 | 1 | 1 | 1 | 5 |
| **179** | (Bunster et al., 2022) | 1 | 0 | 1 | 1 | 1 | 4 |
| **180** | (Burke et al., 2022) | 1 | 1 | 1 | 1 | 1 | 5 |
| **181** | (Cloosterman et al., 2022) | 1 | 1 | 1 | 1 | 1 | 5 |
| **182** | (G. A. Desai & Gruber, 2022) | 1 | 1 | 1 | 1 | 1 | 5 |
| **183** | (Fortune et al., 2022) | 1 | 0 | 1 | 1 | 1 | 4 |
| **184** | (Loudon & Parkerson-Mitchell, 2022) | 1 | 1 | 1 | 1 | 0 | 4 |
| **185** | (Madsen et al., 2022) | 1 | 1 | 1 | 1 | 1 | 5 |
| **186** | (Malisoux et al., 2022) | 1 | 1 | 1 | 1 | 1 | 5 |
| **187** | (Mokwena et al., 2022) | 1 | 1 | 1 | 1 | 1 | 5 |
| **188** | (Ramskov et al., 2022) | 1 | 1 | 1 | 1 | 1 | 5 |
| **189** | (Schmida et al., 2022) | 1 | 1 | 1 | 1 | 1 | 5 |
| **190** | (Suda et al., 2022) | 1 | 1 | 1 | 1 | 1 | 5 |
| **191** | (Swanevelder et al., 2022) | 1 | 1 | 1 | 1 | 1 | 5 |
| **192** | (Toresdahl et al., 2022) | 1 | 0 | 1 | 1 | 1 | 4 |
| **193** | (van Iperen, de Jonge, Gevers, Vos, et al., 2022) | 1 | 1 | 1 | 1 | 1 | 5 |
| **194** | (van Iperen, de Jonge, Gevers, & Vos, 2022) | 0 | 1 | 1 | 1 | 1 | 4 |
| **195** | (Venable et al., 2022) | 1 | 1 | 1 | 1 | 1 | 5 |
| **196** | (Baart et al., 2023) | 1 | 0 | 1 | 1 | 1 | 4 |
| **197** | (Burke et al., 2023) | 1 | 1 | 1 | 1 | 1 | 5 |
| **198** | (Chen et al., 2023) | 1 | 0 | 1 | 1 | 1 | 4 |
| **199** | (Davinelli et al., 2023) | 1 | 1 | 1 | 1 | 1 | 5 |
| **200** | (P. Desai et al., 2023) | 1 | 1 | 1 | 1 | 1 | 5 |
| **201** | (Fokkema et al., 2023) | 1 | 1 | 1 | 1 | 1 | 5 |
| **202** | (Frederico et al., 2023) | 1 | 0 | 1 | 1 | 1 | 4 |
| **203** | (Slabber et al., 2023) | 1 | 1 | 1 | 1 | 1 | 5 |
| **204** | (Van Der Does et al., 2023) | 1 | 1 | 1 | 1 | 1 | 5 |

Appendix F: Years of study publication years (until June 2023)


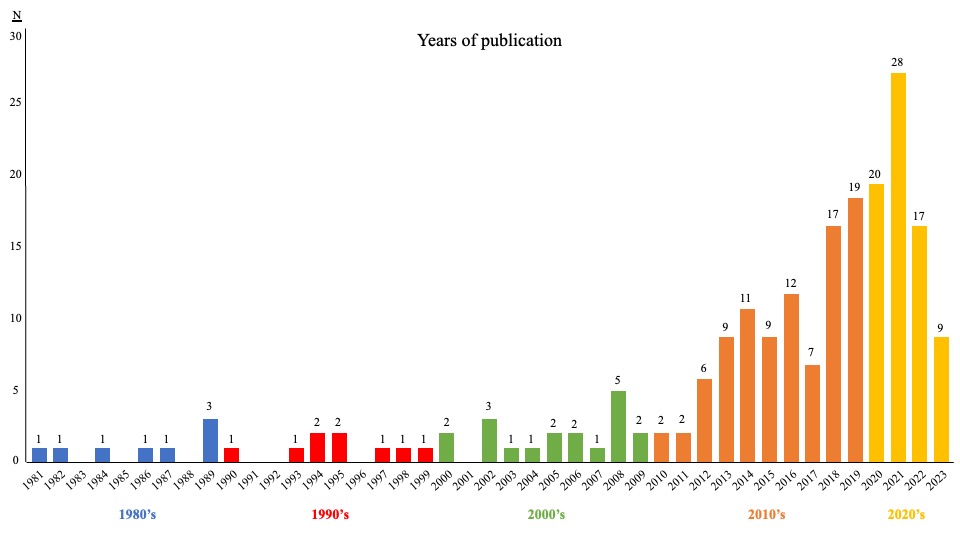


Appendix G: Types of runners (excluding those not specified)


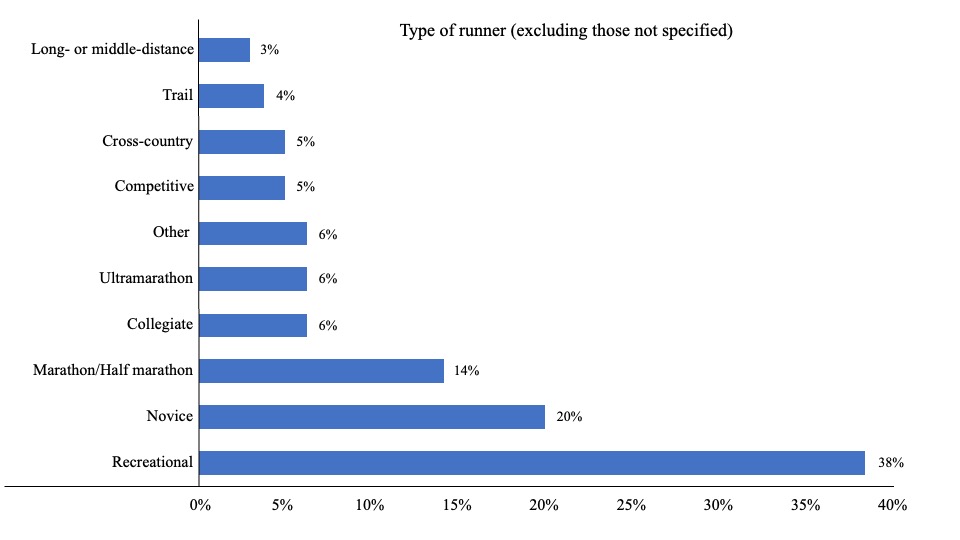


Appendix H: Criteria, descriptors, and sub-descriptors used to define running-related injuries.

|  | **Primary criteria**  ***(% of total studies)*** | **Descriptors**  ***(% of associated criterion)*** | **Sub-descriptors**  ***(% of associated descriptor)*** |
| --- | --- | --- | --- |
| **1** | Physical description  *(94%, n=192)* | Pain *(45%, n=87)* | - |
|  |  | Injury *(28%, n=53)* | - |
|  |  | Physical complaint *(20%, n=39)* | - |
|  |  | Other *(15%, n=29)* | - |
|  |  | Symptom *(7%, n=14)* | - |
|  |  | Problem *(4%, n=7)* | - |
| **2** | Effect on training  *(85%, n=174)* | Training restriction  *(74%, n=128)* | Reduced volume *(55%, n=70)* |
|  |  |  | Reduced intensity *(52%, n=67)* |
|  |  |  | Reduced duration *(41%, n=53)* |
|  |  |  | Reduced frequency *(30%, n=38)* |
|  |  |  | General restriction *(27%, n=35)* |
|  |  |  | Other *(14%, n=18)* |
|  |  |  | Reduced performance *(5%, n=6)* |
|  |  | Time loss  *(51%, n=89)* | Missed training *(100%, n=89)* |
|  |  |  | Missed competition *(4%, n=4)* |
| **3** | Medical intervention  *(36%, n=74)* | Medical attention *(95%, n=70)* | - |
|  |  | Medication *(19%, n=14)* | - |
|  |  | Diagnosis *(7%, n=5)* | - |
|  | **Secondary criteria** | **Descriptors** | **Sub-descriptors** |
| **1** | Cause/Onset of injury  *(75%, n=153)* | Running-related *(96%, n=147)* | - |
|  |  | Overuse *(11%, n=17)* | - |
|  |  | Other *(1%, n=2)* | - |
| **2** | Location  *(72%, n=146)* | Musculoskeletal *(84%, n=123)* | - |
|  |  | Lower limb *(65%, n=95)* | - |
|  |  | Lower back *(35%, n=51)* | - |
|  |  | Other *(9%, n=13)* | - |
| **3** | Social consequences *(3%, n=6)* | Daily life effects *(100%, n=6)* | - |

Note: frequencies and percentages represent the overall number of studies in which each primary criterion, descriptor or sub-descriptor was included (not the frequency or percentage they were used in combination with one another).

Appendix I: Six descriptors associated with the ‘physical description’ criterion.


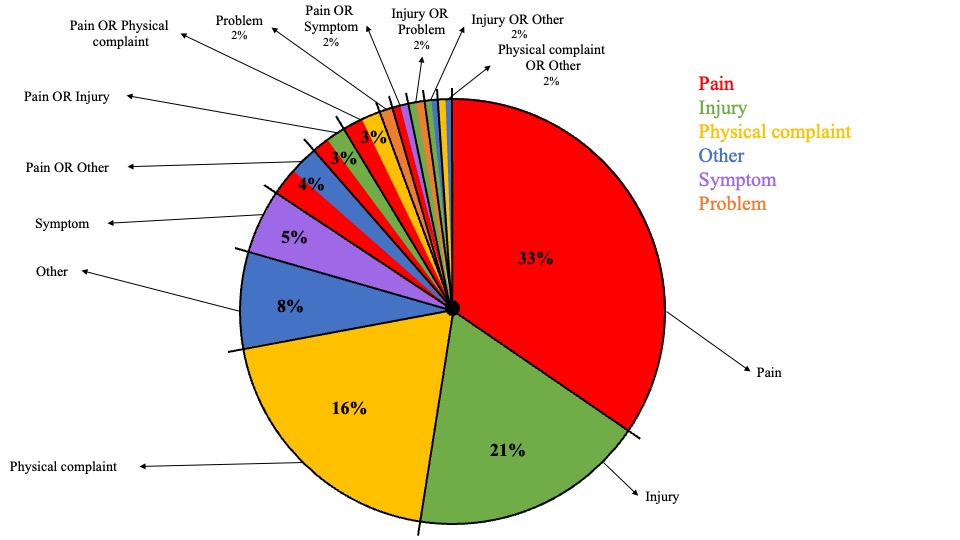


Appendix J: Two descriptors associated with the ‘effect on training’ criterion.


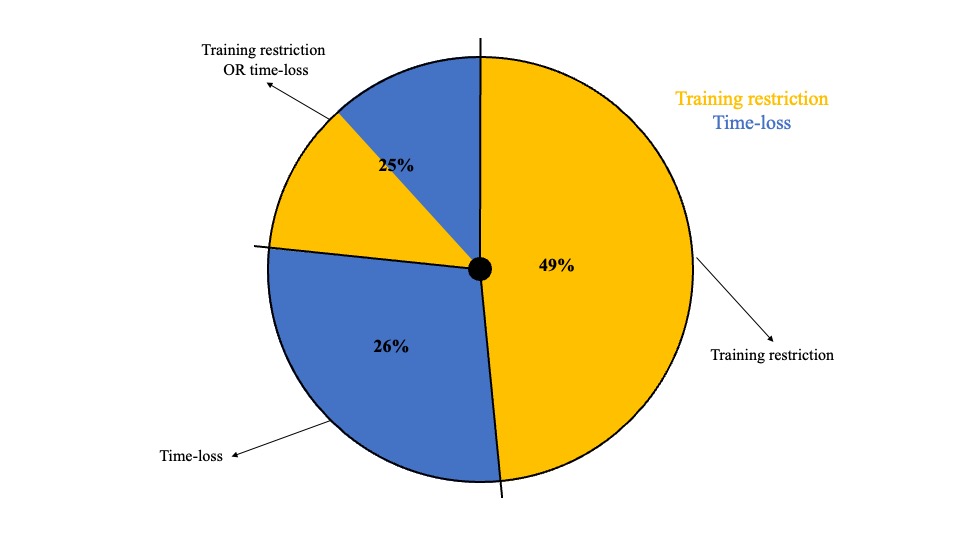


Appendix K: Minimum lengths required for the ‘effect on training’ criterion (i.e., time-loss and training restriction considered together).


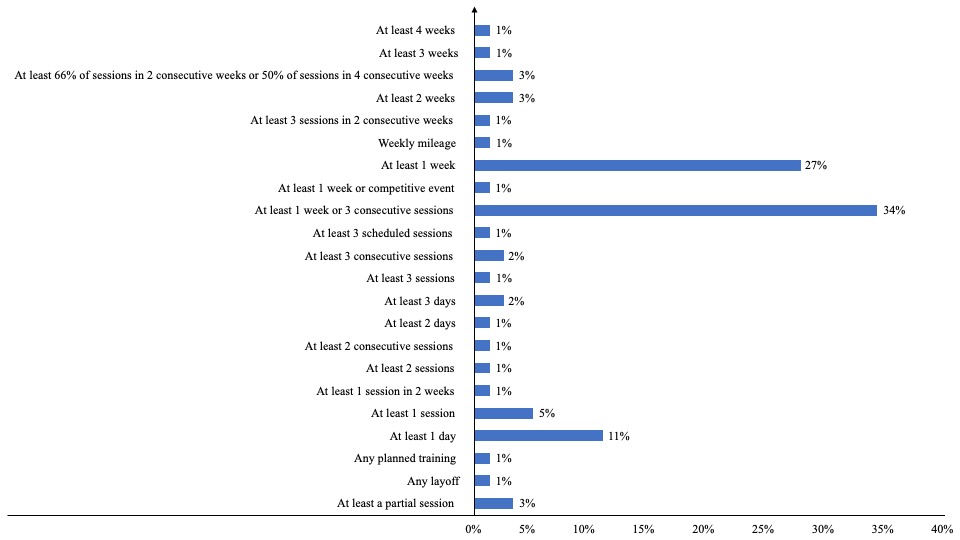


Appendix L: Three descriptors associated with the ‘medical intervention’ criterion.


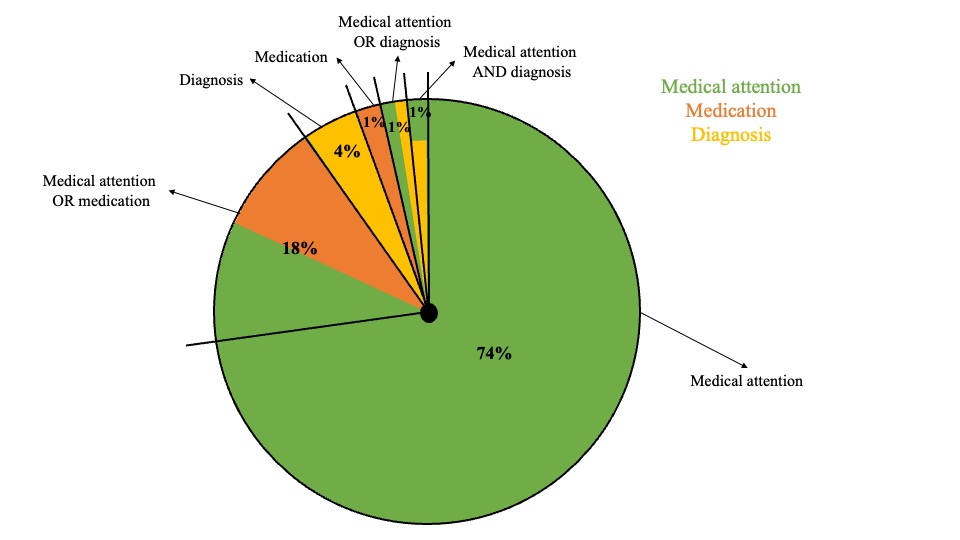


Appendix M: Average incidence rate based on the definition of injury.


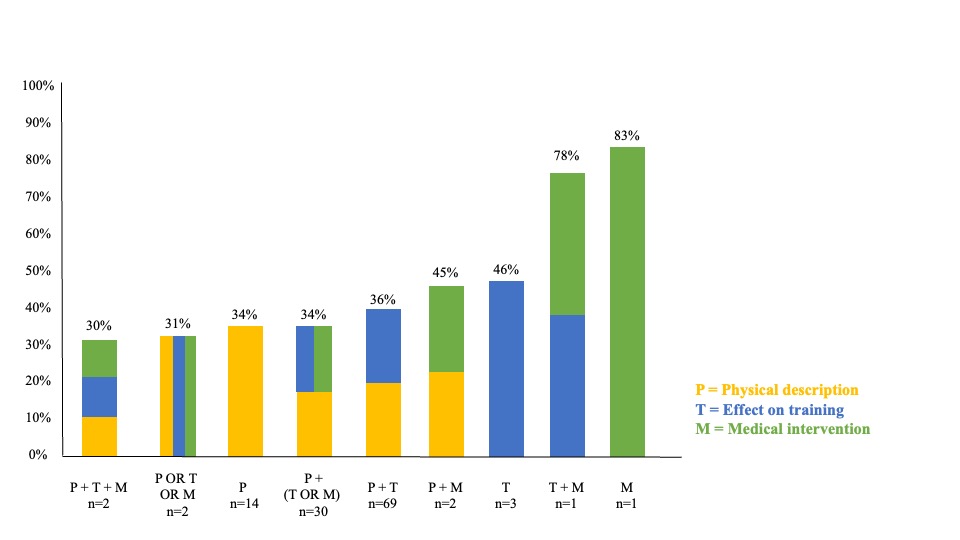


Appendix N: List of articles that directly or indirectly use the consensus definition (Nielsen et al., 2014).

|  | **Study** | **Use of consensus definition** |
| --- | --- | --- |
| **1** | (Besomi et al., 2018) | Direct |
| **2** | (Leppe & Besomi, 2018) | Direct |
| **3** | (Mulvad et al., 2018) | Direct |
| **4** | (Napier et al., 2018) | Direct |
| **5** | (Damsted, Parner, Sørensen, Malisoux, Hulme, et al., 2019) | Direct |
| **6** | (Damsted, Parner, Sørensen, Malisoux, & Nielsen, 2019) | Direct |
| **7** | (Fokkema, de Vos, van Ochten, et al., 2019) | Indirect |
| **8** | (Fokkema, de Vos, Bierma-Zeinstra, et al., 2019) | Indirect |
| **9** | (Onal et al., 2019) | Direct |
| **10** | (Payne et al., 2019) | Indirect |
| **11** | (Perez-Morcillo et al., 2019) | Direct |
| **12** | (Tenforde et al., 2019) | Direct |
| **13** | (Craddock et al., 2020) | Direct |
| **14** | (de Jonge et al., 2020) | Direct |
| **15** | (Fokkema et al., 2020) | Indirect |
| **16** | (Jauhiainen et al., 2020) | Direct |
| **17** | (Johnson et al., 2020) | Direct |
| **18** | (Jungmalm et al., 2020) | Direct |
| **19** | (Letafatkar, Rabiei, & Afshari, 2020) | Direct |
| **20** | (Malisoux et al., 2020) | Direct |
| **21** | (Tenforde et al., 2020) | Direct |
| **22** | (J. Davis & Gruber, 2021) | Direct |
| **23** | (DE OLIVEIRA et al., 2021) | Direct |
| **24** | (P. Desai et al., 2021) | Direct |
| **25** | (Dillon et al., 2021) | Direct |
| **26** | (Hollander et al., 2021) | Direct |
| **27** | (Malisoux et al., 2021) | Direct |
| **28** | (Mousavi et al., 2021) | Direct |
| **29** | (Nakaoka et al., 2021) | Direct |
| **30** | (Quirino et al., 2021) | Direct |
| **31** | (Rhim et al., 2021) | Direct |
| **32** | (Sleeswijk Visser et al., 2021) | Indirect |
| **33** | (Bunster et al., 2022) | Direct |
| **34** | (Burke et al., 2022) | Direct |
| **35** | (Cloosterman et al., 2022) | Direct |
| **36** | (G. A. Desai & Gruber, 2022) | Direct |
| **37** | (Fortune et al., 2022) | Direct |
| **38** | (Malisoux et al., 2022) | Direct |
| **39** | (Schmida et al., 2022) | Direct |
| **40** | (van Iperen, de Jonge, Gevers, Vos, et al., 2022) | Direct |
| **41** | (van Iperen, de Jonge, Gevers, & Vos, 2022) | Direct |
| **42** | (Burke et al., 2023) | Direct |
| **43** | (Chen et al., 2023) | Indirect |
| **44** | (P. Desai et al., 2023) | Direct |

Appendix O: Frequency of injury data capture compared with length of studies.

| **Length of Study** | **Every session (n=16)** | **Every week (n=37)** | **Every 2 weeks (n=15)** |
| --- | --- | --- | --- |
| 3-4 weeks | 6% (n=1) | 19% (n=7) | 13% (n=2) |
| 6 weeks | 19% (n=3) | 24% (n=9) | 47% (n=7) |
| 8-10 weeks | 25% (n=4) | 14% (n=5) | 13% (n-2) |
| 12-15 weeks | - | 5% (n=2) | 13% (n=2) |
| 12 months | 44% (n=7) | 30% (n=11) | 13% (n=2) |
| 18 months | 6% (n=1) | - | - |
| 5 years | - | 5% (n=2) | - |
| Cross-country season | - | 3% (n=1) | - |

**Full reference list:**

Adriaensens, L., Hesselink, A., Fabrie, M., Brugmans, M. J. P., & Verhagen, E. A. L. M. (2014). Effectiveness of an online tailored intervention on determinants and behaviour to prevent running related sports injuries: A randomised controlled trial. *Schweizerische Zeitschrift Fur Sportmedizin Und Sporttraumatologie*, *62*(3), 6–13. Scopus.

Agresta, C., Slobodinsky, M., & Tucker, C. (2014). Functional movement ScreenTM--normative values in healthy distance runners. *International Journal of Sports Medicine*, *35*(14), 1203–1207. https://doi.org/10.1055/s-0034-1382055

Baart, A. M., Terink, R., Naeff, M., Naeff, E., Mensink, M., Alsma, J., Witteman, B. J. M., & Zwerver, J. (2023). Factors associated with lower limb tendinopathy in a large cohort of runners: A survey with a particular focus on nutrition. *BMJ Open Sport & Exercise Medicine*, *9*(2), e001570. https://doi.org/10.1136/bmjsem-2023-001570

Baltich, J., Emery, C. A., Whittaker, J. L., & Nigg, B. M. (2017). Running injuries in novice runners enrolled in different training interventions: A pilot randomized controlled trial. *Scandinavian Journal of Medicine & Science in Sports*, *27*(11), 1372–1383. https://doi.org/10.1111/sms.12743

Begizew, D., Grace, J., & van Heerden, H. (2019). Lower-extremity running-related injuries among 10,000-meter long distance runners in Ethiopia. *JOURNAL OF HUMAN SPORT AND EXERCISE*, *14*(2), 358–373. https://doi.org/10.14198/jhse.2019.142.09

Benca, E., Listabarth, S., Flock, F. K. J., Pablik, E., Fischer, C., Walzer, S. M., Dorotka, R., Windhager, R., & Ziai, P. (2020). Analysis of running-related injuries: The Vienna Stdy. *Journal of Clinical Medicine*, *9*(2), 438.

Bertelsen, M., Hansen, M., Rasmussen, S., & Nielsen, R. (2018). THE START-TO-RUN DISTANCE AND RUNNING-RELATED INJURY AMONG OBESE NOVICE RUNNERS:A RANDOMIZED TRIAL. *INTERNATIONAL JOURNAL OF SPORTS PHYSICAL THERAPY*, *13*(6), 943–955. https://doi.org/10.26603/ijspt20180943

Besomi, M., Leppe, J., Di Silvestre, M. C., & Setchell, J. (2018). SeRUN® study: Development of running profiles using a mixed methods analysis. *PLOS ONE*, *13*(7), e0200389. https://doi.org/10.1371/journal.pone.0200389

Besomi, M., Leppe, J., Mauri-Stecca, M., Hooper, T., & Sizer, P. (2019). Training volume and previous injury as associated factors for running-related injuries by race distance: A cross-sectional study. *JOURNAL OF HUMAN SPORT AND EXERCISE*, *14*(3), 549–559. https://doi.org/10.14198/jhse.2019.143.06

Bishop, G. W., & Fallon, K. E. (1999). Musculoskeletal injuries in a six-day track race: Ultramarathoner’s ankle. *Clinical Journal of Sport Medicine: Official Journal of the Canadian Academy of Sport Medicine*, *9*(4), 216–220. https://doi.org/10.1097/00042752-199910000-00006

Blair, S. N., Kohl, H. W., & Goodyear, N. N. (1987). Rates and Risks for Running and Exercise Injuries: Studies in Three Populations. *Research Quarterly for Exercise and Sport*, *58*(3), 221–228. https://doi.org/10.1080/02701367.1987.10605453

Bovens, A. M., Janssen, G. M., Vermeer, H. G., Hoeberigs, J. H., Janssen, M. P., & Verstappen, F. T. (1989). Occurrence of running injuries in adults following a supervised training program. *International Journal of Sports Medicine*, *10 Suppl 3*, S186-190. https://doi.org/10.1055/s-2007-1024970

Bredeweg, S. W., Kluitenberg, B., Bessem, B., & Buist, I. (2013). Differences in kinetic variables between injured and noninjured novice runners. *Journal of Science and Medicine in Sport*, *16*(3), 205–210.

Bredeweg, S. W., Zijlstra, S., Bessem, B., & Buist, I. (2012). The effectiveness of a preconditioning programme on preventing running-related injuries in novice runners: A randomised controlled trial. *British Journal of Sports Medicine*, *46*(12), 865–870. https://doi.org/10.1136/bjsports-2012-091397

Brund, R. B. K., Rasmussen, S., Nielsen, R. O., Kersting, U. G., Laessoe, U., & Voigt, M. (2017). Medial shoe-ground pressure and specific running injuries: A 1-year prospective cohort study. *Journal of Science and Medicine in Sport*, *20*(9), 830–834. https://doi.org/10.1016/j.jsams.2017.04.001

Brund, R., Nielsen, R., Parner, E., Rasmussen, S., & Voigt, M. (2019). Changes in the running-related injury incidence rate ratio in a 1000-km explorative prospective cohort study involving two unspecific shoe changes. *FOOTWEAR SCIENCE*, *11*(2), 63–70. https://doi.org/10.1080/19424280.2018.1529063

Buist, I., Bredeweg, S. W., Bessem, B., van Mechelen, W., Lemmink, K. A. P. M., & Diercks, R. L. (2010). Incidence and risk factors for running-related injuries during preparation for a 4-mile recreational running event. *British Journal of Sports Medicine*, *44*(8), 598–604.

Buist, I., Bredeweg, S. W., Lemmink, K. A. P. M., van Mechelen, W., & Diercks, R. L. (2010). Predictors of running-related injuries in novice runners enrolled in a systematic training program: A prospective cohort study. *The American Journal of Sports Medicine*, *38*(2), 273–280. https://doi.org/10.1177/0363546509347985

Buist, I., Bredeweg, S. W., van Mechelen, W., Lemmink, K. A. P. M., Pepping, G.-J., & Diercks, R. L. (2008). No effect of a graded training program on the number of running-related injuries in novice runners: A randomized controlled trial. *The American Journal of Sports Medicine*, *36*(1), 33–39. https://doi.org/10.1177/0363546507307505

Bunster, J., Martinez, M., Mauri-Stecca, M., Leppe, J., Nelson, E., Heiderscheit, B., & Besomi, M. (2022). Cross-cultural adaptation and evaluation of the psychometric properties of the University of Wisconsin Running Injury and Recovery Index questionnaire in Spanish (UWRI-S). *PHYSICAL THERAPY IN SPORT*, *55*, 289–295. https://doi.org/10.1016/j.ptsp.2022.05.007

Burke, A., Dillon, S., O’Connor, S., Whyte, E. F., Gore, S., & Moran, K. A. (2023). Aetiological Factors of Running-Related Injuries: A 12 Month Prospective ‘Running Injury Surveillance Centre’ (RISC) Study. *Sports Medicine - Open*, *9*(1), 46. https://doi.org/10.1186/s40798-023-00589-1

Burke, A., Dillon, S., O’Connor, S., Whyte, E., Gore, S., & Moran, K. A. (2022). Comparison of impact accelerations between injury-resistant and recently injured recreational runners. *PloS ONE*, *17*(9), e0273716.

Cahanin, R., Jefferson, J., Flynn, T., & Goyeneche, N. (2019). ILIAC CREST HEIGHT DIFFERENCE AND OTHER RUNNING-RELATED VARIABLES’ RELATIONSHIP WITH RUNNING INJURY. *INTERNATIONAL JOURNAL OF SPORTS PHYSICAL THERAPY*, *14*(6), 957–966. https://doi.org/10.26603/ijspt20190957

Chan, Z. Y. S., Zhang, J. H., Au, I. P. H., An, W. W., Shum, G. L. K., Ng, G. Y. F., & Cheung, R. T. H. (2018). Gait Retraining for the Reduction of Injury Occurrence in Novice Distance Runners: 1-Year Follow-up of a Randomized Controlled Trial. *The American Journal of Sports Medicine*, *46*(2), 388–395. https://doi.org/10.1177/0363546517736277

Chang, W.-L., Shih, Y.-F., & Chen, W.-Y. (2012). Running injuries and associated factors in participants of ING Taipei Marathon. *Physical Therapy in Sport : Official Journal of the Association of Chartered Physiotherapists in Sports Medicine*, *13*(3), 170–174. https://doi.org/10.1016/j.ptsp.2011.08.001

Chen, W., Cloosterman, K. L. A., Bierma-Zeinstra, S. M. A., van Middelkoop, M., & de Vos, R.-J. (2023). Epidemiology of insertional and midportion Achilles tendinopathy in runners: A prospective cohort study. *Journal of Sport and Health Science*. cmedm. https://doi.org/10.1016/j.jshs.2023.03.007

Chorley, J. N., Cianca, J. C., Divine, J. G., & Hew, T. D. (2002). Baseline injury risk factors for runners starting a marathon training program. *Clinical Journal of Sport Medicine : Official Journal of the Canadian Academy of Sport Medicine*, *12*(1), 18–23. https://doi.org/10.1097/00042752-200201000-00007

Cloosterman, K. L. A., Fokkema, T., de Vos, R.-J., Visser, E., Krastman, P., IJzerman, J., Koes, B. W., Verhaar, J. A. N., Bierma-Zeinstra, S. M. A., & van Middelkoop, M. (2022). Educational online prevention programme (the SPRINT study) has no effect on the number of running-related injuries in recreational runners: A randomised-controlled trial. *British Journal of Sports Medicine*, *56*(12), 676–682. https://doi.org/10.1136/bjsports-2021-104539

Costa, M. E. F., Fonseca, J. B., de Oliveira, A. I. S., Cabral, K. D. A., de Araújo, M. D. G. R., & Ferreira, A. P. L. (2020). Prevalence and factors associated with injuries in recreational runners: A cross-sectional study [Prevalência e fatores associados às lesões em corredores amadores: Um estudo transversal]. *Revista Brasileira de Medicina Do Esporte*, *26*(3), 215–219. https://doi.org/10.1590/1517-869220202603190923

Craddock, N., Buchholtz, K., & Burgess, T. L. (2020). Does a greater training load increase the risk of injury and illness in ultramarathon runners? : A prospective, descriptive, longitudinal design. *South African Journal of Sports Medicine*, *32*(1), v32i1a8559. cmedm. https://doi.org/10.17159/2078-516X/2020/v32i1a8559

Dallinga, J., van Rijn, R., Stubbe, J., & Deutekom, M. (2019). Injury incidence and risk factors: A cohort study of 706 8-km or 16-km recreational runners. *BJM Open Sport & Exercise Medicine*, *5*(1), e000489.

Damsted, C., Parner, E. T., Sørensen, H., Malisoux, L., Hulme, A., & Nielsen, R. Ø. (2019). The Association Between Changes in Weekly Running Distance and Running-Related Injury: Preparing for a Half Marathon. *The Journal of Orthopaedic and Sports Physical Therapy*, *49*(4), 230–238. https://doi.org/10.2519/jospt.2019.8541

Damsted, C., Parner, E. T., Sørensen, H., Malisoux, L., & Nielsen, R. O. (2019). ProjectRun21: Do running experience and running pace influence the risk of running injury-A 14-week prospective cohort study. *Journal of Science and Medicine in Sport*, *22*(3), 281–287. https://doi.org/10.1016/j.jsams.2018.08.014

Davinelli, S., Intrieri, M., Ali, S., Righetti, S., Mondazzi, L., Scapagnini, G., & Corbi, G. (2023). Omega-3 index and AA/EPA ratio as biomarkers of running-related injuries: An observational study in recreational runners. *European Journal of Sport Science*, *23*(1), 134–142. https://doi.org/10.1080/17461391.2021.1998643

Davis, I. S., Bowser, B. J., & Mullineaux, D. R. (2016). Greater vertical impact laoding in female runners with medically diagnosed injuries: A prospective investigation. *British Journal of Sports Medicine*, *50*(14), 887–892.

Davis, J., & Gruber, A. (2021). Leg Stiffness, Joint Stiffness, and Running-Related Injury: Evidence From a Prospective Cohort Study. *ORTHOPAEDIC JOURNAL OF SPORTS MEDICINE*, *9*(5). https://doi.org/10.1177/23259671211011213

de Jonge, J., Balk, Y. A., & Taris, T. W. (2020). Mental Recovery and Running-Related Injuries in Recreational Runners: The Moderating Role of Passion for Running. *International Journal of Environmental Research and Public Health*, *17*(3). https://doi.org/10.3390/ijerph17031044

DE OLIVEIRA, B. G., TEIXEIRA, R., BORTOLIFUNEZ, E. I., RODRIGUEZ-AÑEZ, C. R., NAHHAS RODACKI, C. D. L., & FERMINO, R. C. (2021). Injury in street runners: Prevalence and associated factors. *Journal of Physical Education & Sport*, *21*(1), 21–28. s3h.

Desai, G. A., & Gruber, A. H. (2021). Segment coordination and variability among prospectively injured and uninjured runners. *Journal of Sports Sciences*, *39*(1), 38–47. https://doi.org/10.1080/02640414.2020.1804519

Desai, G. A., & Gruber, A. H. (2022). Bilateral differences in coordination variability among injured and uninjured runners: A prospective study. *Journal of Biomechanics*, *132*, 110938. https://doi.org/10.1016/j.jbiomech.2021.110938

Desai, P., Jungmalm, J., Borjesson, M., Karlsson, J., & Grau, S. (2021). Recreational runners with a history of injury are twice as likely to sustain a running-related injury as runners with no histroy of injury: A 1-year prospective cohort study. *The Journal of Orthopaedic and Sports Physical Therapy*, *51*(3), 144–150.

Desai, P., Jungmalm, J., Börjesson, M., Karlsson, J., & Grau, S. (2023). Effectiveness of an 18-week general strength and foam-rolling intervention on running-related injuries in recreational runners. *Scandinavian Journal of Medicine & Science in Sports*, *33*(5), 766–775. https://doi.org/10.1111/sms.14313

Dijkhuis, T. B., Otter, R., Aiello, M., Velthuijsen, H., & Lemmink, K. (2020). Increase in the Acute:Chronic Workload Ratio relates to Injury Risk in Competitive Runners. *International Journal of Sports Medicine*, *41*(11), 736–743. https://doi.org/10.1055/a-1171-2331

Dillon, S., Burke, A., Whyte, E. F., O’Connor, S., Gore, S., & Moran, K. A. (2021). Do Injury-Resistant Runners Have Distinct Differences in Clinical Measures Compared with Recently Injured Runners? *Medicine and Science in Sports and Exercise*, *53*(9), 1807–1817. https://doi.org/10.1249/MSS.0000000000002649

Dubois, B., Esculier, J.-F., Frémont, P., Moore, L., & Richards, C. (2015). Effects of minimalist and traditional running shoes on injury rates: A pilot randomised controlled trial. *Footwear Science*, *7*(3), 159–164. Scopus. https://doi.org/10.1080/19424280.2015.1049300

Dudley, R. I., Pamukoff, D. N., Lynn, S. K., Kersey, R. D., & Noffal, G. J. (2017). A prospective comparison of lower extremity kinematics and kinetics between injured and non-injured collegiate cross country runners. *Human Movement Science*, *52*, 197–202. https://doi.org/10.1016/j.humov.2017.02.007

Duffey, M. J., Martin, D. F., Cannon, D. W., Craven, T., & Messier, S. P. (2000). Etiologic factors associated with anterior knee pain in distance runners. *Medicine and Science in Sports and Exercise*, *32*(11), 1825–1832. https://doi.org/10.1097/00005768-200011000-00003

Ellapen, T. J., Satyendra, S., Morris, J., & van Heerden, H. J. (2013). Common running musculoskeletal injuries among recreational half-marathon runners in KwaZulu-Natal. *South Africian Sports Medicine Association*, *25*(2), 39.

Eskofier, B. M., Kraus, M., Worobets, J. T., Stefanyshyn, D. J., & Nigg, B. M. (2012). Pattern classification of kinematic and kinetic running data to distinguish gender, shod/barefoot and injury groups with feature ranking. *Computer Methods in Biomechanics and Biomedical Engineering*, *15*(5), 467–474. https://doi.org/10.1080/10255842.2010.542153

Ferreira, A. C., Dias, J. M. C., Fernandes, R. M., Sabino, G. S., dos Anjos, M. T. S., & Felício, D. C. (2012). Prevalence and associated risks of injury in amateur street runners from Belo Horizonte, MG. *Revista Brasileira de Medicina Do Esporte*, *18*(4), 252–255. Scopus. https://doi.org/10.1590/S1517-86922012000400007

Fields, K. B., Delaney, M., & Hinkle, J. S. (1990). A prospective study of type A behavior and running injuries. *The Journal of Family Practice*, *30*(4), 425–429.

Fokkema, T., de Vos, R.-J., Bierma-Zeinstra, S. M. A., & van Middelkoop, M. (2019). Opinions, Barriers, and Facilitators of Injury Prevention in Recreational Runners. *The Journal of Orthopaedic and Sports Physical Therapy*, *49*(10), 736–742. https://doi.org/10.2519/jospt.2019.9029

Fokkema, T., de Vos, R.-J., van Ochten, J. M., Verhaar, J. A. N., Davis, I. S., Bindels, P. J. E., Bierma-Zeinstra, S. M. A., & van Middelkoop, M. (2019). Online multifactorial prevention programme has no effect on the number of running-related injuries: A randomised controlled trial. *British Journal of Sports Medicine*, *53*(23), 1479–1485. https://doi.org/10.1136/bjsports-2018-099744

Fokkema, T., van Damme, A. A. D. N., Fornerod, M. W. J., de Vos, R.-J., Bierma-Zeinstra, S. M. A., & van Middelkoop, M. (2020). Training for a (half-)marathon: Training volume and longest endurance run related to performance and running injuries. *Scandinavian Journal of Medicine & Science in Sports*, *30*(9), 1692–1704. https://doi.org/10.1111/sms.13725

Fokkema, T., Varkevisser, N., de Vos, R.-J., Bierma-Zeinstra, S. M. A., & van Middelkoop, M. (2023). Factors Associated With Running-Related Injuries in Recreational Runners With a History of Running Injuries. *Clinical Journal of Sport Medicine : Official Journal of the Canadian Academy of Sport Medicine*, *33*(1), 61–66. https://doi.org/10.1097/JSM.0000000000001076

Fortune, A., Sims, J., Rhodes, S., & Ampat, G. (2022). Does orthotics use improve comfort, speed and injury rate during running? Preliminary analysis of a randomised control trial. *WORLD JOURNAL OF ORTHOPEDICS*, *13*(7), 652–661. https://doi.org/10.5312/wjo.v13.i7.652

Franke, T., de Vet, H., & Huisstede, B. (2021). Minimally important change and smallest detectable change of the OSTRC questionnaire in half- and full-marathon runners. *SCANDINAVIAN JOURNAL OF MEDICINE & SCIENCE IN SPORTS*, *31*(5), 1048–1058. https://doi.org/10.1111/sms.13885

Franke, T. P. C., Backx, F. J. G., & Huisstede, B. M. A. (2019). Running Themselves Into the Ground? Incidence, Prevalence, and Impact of Injury and Illness in Runners Preparing for a Half or Full Marathon. *The Journal of Orthopaedic and Sports Physical Therapy*, *49*(7), 518–528. https://doi.org/10.2519/jospt.2019.8473

Frederico, R. A., Santos, T. R. T., Okai-Nóbrega, L. A., Ocarino, J. M., Souza, T. R., & Fonseca, S. T. (2023). Runners with a history of shank and foot injury: Interactions among local musculoskeletal factors, age, and running experience. *Physical Therapy in Sport*, *62*, 1–9. https://doi.org/10.1016/j.ptsp.2023.05.001

Fuller, J. T., Thewlis, D., Buckley, J. D., Brown, N. A. T., Hamill, J., & Tsiros, M. D. (2017). Body Mass and Weekly Training Distance Influence the Pain and Injuries Experienced by Runners Using Minimalist Shoes: A Randomized Controlled Trial. *The American Journal of Sports Medicine*, *45*(5), 1162–1170. https://doi.org/10.1177/0363546516682497

Gajardo-Burgos, R., Monrroy-Uarac, M., Barria-Pailaquilen, R., Norambuena-Noches, Y., van Rensburg, D., Bascour-Sandoval, C., & Besomi, M. (2021). Frequency of Injury and Illness in the Final 4 Weeks before a Trail Running Competition. *INTERNATIONAL JOURNAL OF ENVIRONMENTAL RESEARCH AND PUBLIC HEALTH*, *18*(10). https://doi.org/10.3390/ijerph18105431

Gerlach, K., Burton, H., Dorn, J., Leddy, J., & Horvath, P. (2008). Fat intake and injury in female runners. *JOURNAL OF THE INTERNATIONAL SOCIETY OF SPORTS NUTRITION*, *5*. https://doi.org/10.1186/1550-2783-5-1

Gerlach, K. E., White, S. C., Burton, H. W., Dorn, J. M., Leddy, J. J., & Horvath, P. J. (2005). Kinetic changes with fatigue and relationship to injury in female runners. *Medicine and Science in Sports and Exercise*, *37*(4), 657–663. https://doi.org/10.1249/01.mss.0000158994.29358.71

Graham, S. M., Martindale, R. J. J., McKinley, M., Connaboy, C., Andronikos, G., & Susmarski, A. (2021). The examination of mental toughness, sleep, mood and injury rates in an Arctic ultra-marathon. *European Journal of Sport Science*, *21*(1), 100–106. s3h.

Gruber, A., McDonnell, J., Davis, J., Vollmar, J., Harezlak, J., & Paquette, M. (2021). Monitoring Gait Complexity as an Indicator for Running-Related Injury Risk in Collegiate Cross-Country Runners: A Proof-of-Concept Study. *FRONTIERS IN SPORTS AND ACTIVE LIVING*, *3*. https://doi.org/10.3389/fspor.2021.630975

Gutierrez-Hellin, J., Baltazar-Martins, G., Aguilar-Navarro, M., Ruiz-Moreno, C., Olivan, J., & Del Coso, J. (2021). Effect of ACTN3 R577X Genotype on Injury Epidemiology in Elite Endurance Runners. *GENES*, *12*(1). https://doi.org/10.3390/genes12010076

Hamstra-Wright, K. L., Coumbe-Lilley, J. E., Kim, H., McFarland, J. A., & Huxel Bliven, K. C. (2013). The influence of training and mental skills preparation on injury incidence and performance in marathon runners. *Journal of Strength and Conditioning Research*, *27*(10), 2828–2835. https://doi.org/10.1519/JSC.0b013e31828a4733

Hein, T., Janssen, P., Wagner-Fritz, U., Haupt, G., & Grau, S. (2014). Prospective analysis of intrinsic and extrinsic risk factors on the development of Achilles tendon pain in runners: Risk factors for Achilles tendon pain. *Scandinavian Journal of Medicine & Science in Sports*, *24*(3), e201–e212. https://doi.org/10.1111/sms.12137

Hendricks, C., & Phillips, J. (2013). Prevalence and incidence rate of injuries in runners at a local athletic club in Cape Town. *South African Journal of Physiotherapy*, *69*(3), 33–37. https://doi.org/10.4102/sajp.v69i3.31

Hesar NG, Van Ginckel A, Cools A, Peersman W, Roosen P, De Clercq D, & Witvrouw E. (2009). A prospective study on gait-related intrinsic risk factors for lower leg overuse injuries. *British Journal of Sports Medicine*, *43*(14), 1057–1061. s3h.

Hespanhol Junior, L. C., Costa, L. O. P., Carvalho, A. C. A., & Lopes, A. D. (2012). A description of training characteristics and its association with previous musculoskeletal injuries in recreational runners: A cross-sectional study. *Revista brasileira de fisioterapia (Sao Carlos (Sao Paulo, Brazil))*, *16*(1), 46–53.

Hespanhol, L. C., van Mechelen, W., & Verhagen, E. (2018). Effectiveness of online tailored advice to prevent running-related injuries and promote preventive behaviour in Dutch trail runners: A pragmatic randomised controlled trial. *British Journal of Sports Medicine*, *52*(13), 851–858. https://doi.org/10.1136/bjsports-2016-097025

Hespanhol, L., Costa, L., & Lopes, A. (2013). Previous injuries and some training characteristics predict running-related injuries in recreational runners: A prospective cohort study. *JOURNAL OF PHYSIOTHERAPY*, *59*(4), 263–269. https://doi.org/10.1016/S1836-9553(13)70203-0

Hespanhol, L., de Carvalho, A., Costa, L., & Lopes, A. (2016). Lower limb alignment characteristics are not associated with running injuries in runners: Prospective cohort study. *EUROPEAN JOURNAL OF SPORT SCIENCE*, *16*(8), 1137–1144. https://doi.org/10.1080/17461391.2016.1195878

Hespanhol, L., Huisstede, B., Smits, D., Kluitenberg, B., van der Worp, H., van Middelkoop, M., Hartgens, F., & Verhagen, E. (2016). The NLstart2run study: Economic burden of running-related injuries in novice runners participating in a novice running program. *JOURNAL OF SCIENCE AND MEDICINE IN SPORT*, *19*(10), 800–804. https://doi.org/10.1016/j.jsams.2015.12.004

Hespanhol, L., Vallio, C. S., van Mechelen, W., & Verhagen, E. (2021). Can we explain running-related injury preventive behavior? A path analysis. *Brazilian Journal of Physical Therapy*, *25*(5), 601–609. https://doi.org/10.1016/j.bjpt.2021.04.007

Hespanhol, L., van Mechelen, W., Postuma, E., & Verhagen, E. (2016). Health and economic burden of running-related injuries in runners training for an event: A prospective cohort study. *SCANDINAVIAN JOURNAL OF MEDICINE & SCIENCE IN SPORTS*, *26*(9), 1091–1099. https://doi.org/10.1111/sms.12541

Hespanhol, L., van Mechelen, W., & Verhagen, E. (2017). Health and Economic Burden of Running-Related Injuries in Dutch Trailrunners: A Prospective Cohort Study. *SPORTS MEDICINE*, *47*(2), 367–377. https://doi.org/10.1007/s40279-016-0551-8

Hjerrild, M., Videbaek, S., Theisen, D., Malisoux, L., & Oestergaard Nielsen, R. (2018). How (not) to interpret a non-causal association in sports injury science. *Physical Therapy in Sport : Official Journal of the Association of Chartered Physiotherapists in Sports Medicine*, *32*, 121–125. https://doi.org/10.1016/j.ptsp.2018.05.009

Hoffman, M. D., & Fogard, K. (2011). Factors related to successful completion of a 161-km ultramarathon. *International Journal of Sports Physiology and Performance*, *6*(1), 25–37. cmedm. https://doi.org/10.1123/ijspp.6.1.25

Hoffman, M. D., & Krishnan, E. (2014). Health and exercise-related medical issues among 1,212 ultramarathon runners: Baseline findings from the Ultrarunners Longitudinal TRAcking (ULTRA) Study. *PloS One*, *9*(1), e83867. https://doi.org/10.1371/journal.pone.0083867

Hofstede, H., Franke, T. P. C., Van Eijk, R. P. A., Backx, F. J. G., Kemler, E., & Huisstede, B. M. A. (2020). In training for a marathon: Runners and running-related injury prevention. *Physical Therapy in Sport*, *41*, 80–86. https://doi.org/10.1016/j.ptsp.2019.11.006

Hollander, K., Johnson, C. D., Outerleys, J., & Davis, I. S. (2021). Multifactorial Determinants of Running Injury Locations in 550 Injured Recreational Runners. *Medicine and Science in Sports and Exercise*, *53*(1), 102–107. https://doi.org/10.1249/MSS.0000000000002455

Holmes, H., Monaghan, P., Strunk, K., Paquette, M., & Roper, J. (2021). Changes in Training, Lifestyle, Psychological and Demographic Factors, and Associations With Running-Related Injuries During COVID-19. *FRONTIERS IN SPORTS AND ACTIVE LIVING*, *3*. https://doi.org/10.3389/fspor.2021.637516

Hootman, J. M., Macera, C. A., Ainsworth, B. E., Martin, M., Addy, C. L., & Blair, S. N. (2002). Predictors of lower extremity injury among recreationally active adults. *Clinical Journal of Sport Medicine : Official Journal of the Canadian Academy of Sport Medicine*, *12*(2), 99–106. https://doi.org/10.1097/00042752-200203000-00006

Hotta, T., Nishiguchi, S., Fukutani, N., Tashiro, Y., Adachi, D., Morino, S., Shirooka, H., Nozaki, Y., Hirata, H., Yamaguchi, M., & Aoyama, T. (2015). Functional Movement Screen for Predicting Running Injuries in 18- to 24-Year-Old Competitive Male Runners. *Journal of Strength and Conditioning Research*, *29*(10), 2808–2815. https://doi.org/10.1519/JSC.0000000000000962

Hutson, M. A. (1984). Medical implications of ultra marathon running: Observations on a six day track race. *British Journal of Sports Medicine*, *18*(1), 44–45. https://doi.org/10.1136/bjsm.18.1.44

Jacobs, S. J., & Berson, B. L. (1986). Injuries to runners: A study of entrants to a 10,000 meter race. *American Journal of Sports Medicine*, *14*(2), 151–155.

Jakobsen, B. W., Krøner, K., Schmidt, S. A., & Kjeldsen, A. (1994). Prevention of injuries in long-distance runners. *Knee Surgery, Sports Traumatology, Arthroscopy : Official Journal of the ESSKA*, *2*(4), 245–249. https://doi.org/10.1007/BF01845597

Jauhiainen, S., Pohl, A. J., Äyrämö, S., Kauppi, J., & Ferber, R. (2020). A hierarchical cluster analysis to determine whether injured runners exhibit similar kinematic gait patterns. *Scandinavian Journal of Medicine & Science in Sports*, *30*(4), 732–740. https://doi.org/10.1111/sms.13624

Johnson, C. D., Tenforde, A. S., Outerleys, J., Reilly, J., & Davis, I. S. (2020). Impact-Related Ground Reaction Forces Are More Strongly Associated With Some Running Injuries Than Others. *The American Journal of Sports Medicine*, *48*(12), 3072–3080. https://doi.org/10.1177/0363546520950731

Jungmalm, J., Nielsen, R., Desai, P., Karlsson, J., Hein, T., & Grau, S. (2020). Associations between biomechanical and clinical/anthropometrical factors and running-related injuries among recreational runners: A 52-week prospective cohort study. *INJURY EPIDEMIOLOGY*, *7*(1). https://doi.org/10.1186/s40621-020-00237-2

Kemler, E., Blokland, D., Backx, F., & Huisstede, B. (2018). Differences in injury risk and characteristics of injuries between novice and experienced runners over a 4-year period. *The Physician and Sports Medicine*, *46*(6), 485–491. https://doi.org/. doi: 10.1080/00913847.2018.1507410

Kemler, E., Romeijn, K., Vriend, I., & Huisstede, B. (2018). The relationship between the use of running applications and running-related injuries. *The Physician and Sportsmedicine*, *46*(1), 73–77. https://doi.org/10.1080/00913847.2018.1412812

Kerr, Z. Y., Kroshus, E., Grant, J., Parsons, J. T., Folger, D., Hayden, R., & Dompier, T. P. (2016). Epidemiology of National Collegiate Athletic Association Men’s and Women’s Cross-Country Injuries, 2009-2010 Through 2013-2014. *Journal of Athletic Training*, *51*(1), 57–64. https://doi.org/10.4085/1062-6050-51.1.10

Kliethermes, S. A., Stiffler-Joachim, M. R., Wille, C. M., Sanfilippo, J. L., Zavala, P., & Heiderscheit, B. C. (2021). Lower step rate is associated with a higher risk of bone stress injury: A prospective study of collegiate cross country runners. *British Journal of Sports Medicine*, *55*(15), 851–856. https://doi.org/10.1136/bjsports-2020-103833

Kluitenberg, B., van der Worp, H., Huisstede, B. M. A., Hartgens, F., Diercks, R., Verhagen, E., & van Middelkoop, M. (2016). The NLstart2run study: Training-related factors associated with running-related injuries in novice runners. *Journal of Science and Medicine in Sport*, *19*(8), 642–646. https://doi.org/10.1016/j.jsams.2015.09.006

Kluitenberg, B., van Middelkoop, M., Smits, D. W., Verhagen, E., Hartgens, F., Diercks, R., & van der Worp, H. (2015). The NLstart2run study: Incidence and risk factors of running-related injuries in novice runners. *Scandinavian Journal of Medicine & Science in Sports*, *25*(5), e515-523. https://doi.org/10.1111/sms.12346

Knobloch, K., Yoon, U., & Vogt, P. M. (2008). Acute and overuse injuries correlated to hours of training in master running athletes. *Foot & Ankle International*, *29*(7), 671–676. https://doi.org/10.3113/FAI.2008.0671

Koech, R. C., Olivier, B., & Tawa, N. (2021). A prevalence of running-related injuries among professional endurance runners in the Rift Valley, Kenya. *South African Journal of Sports Medicine*, *33*(1), v33i1a10690. cmedm. https://doi.org/10.17159/2078-516X/2021/v33i1a10690

Koplan, J. P. (1982). An Epidemiologic Study of the Benefits and Risks of Running. *JAMA: The Journal of the American Medical Association*, *248*(23), 3118. https://doi.org/10.1001/jama.1982.03330230030026

Koplan, J. P., Rothenberg, R. B., & Jones, E. L. (1995). The natural history of exercise: A 10-yr follow-up of a cohort of runners. *Medicine and Science in Sports and Exercise*, *27*(8), 1180–1184. cmedm.

Leppe, J., & Besomi, M. (2018). Recent Versus Old Previous Injury and Its Association with Running-Related Injuries During Competition by SeRUN (R) Running Profiles: A Cross-sectional Study. *SPORTS MEDICINE-OPEN*, *4*. https://doi.org/10.1186/s40798-018-0164-x

Letafatkar, A., Rabiei, P., & Afshari, M. (2020). Effect of neuromuscular training augmented with knee valgus control instructions on lower limb biomechanics of male runners. *Physical Therapy in Sport : Official Journal of the Association of Chartered Physiotherapists in Sports Medicine*, *43*, 89–99. https://doi.org/10.1016/j.ptsp.2020.02.009

Letafatkar, A., Rabiei, P., Farivar, N., & Alamouti, G. (2020). Long-term efficacy of conditioning training program combined with feedback on kinetics and kinematics in male runners. *Scandinavian Journal of Medicine & Science in Sports*, *30*(3), 429–441. https://doi.org/10.1111/sms.13587

Linton, L., & Valentin, S. (2018). Running with injury: A study of UK novice and recreational runners and factor associated with running related injury. *Journal of Science and Medicine in Sport*, *21*(12), 1221–1225.

Loudon, J., & Parkerson-Mitchell, A. (2022). Training Habits and Injury Rate in Masters Female Runners. *INTERNATIONAL JOURNAL OF SPORTS PHYSICAL THERAPY*, *17*(3), 501–507. https://doi.org/10.26603/001c.32374

Luedke, L., & Rauh, M. (2021). Factors Associated With Self-Selected Step Rates Between Collegiate and High School Cross Country Runners. *FRONTIERS IN SPORTS AND ACTIVE LIVING*, *2*. https://doi.org/10.3389/fspor.2020.628348

Lun, V. (2004). Relation between running injury and static lower limb alignment in recreational runners. *British Journal of Sports Medicine*, *38*(5), 576–580. https://doi.org/10.1136/bjsm.2003.005488

Macera, C. A. (1989). Predicting Lower-Extremity Injuries Among Habitual Runners. *Archives of Internal Medicine*, *149*(11), 2565. https://doi.org/10.1001/archinte.1989.00390110117026

Madsen, A., Sharififar, S., Oberhaus, J., Vincent, K. R., & Vincent, H. K. (2022). Anxiety state impact on recovery of runners with lower extremity injuries. *PloS One*, *17*(12), e0278444. https://doi.org/10.1371/journal.pone.0278444

Malisoux, L., Chambon, N., Delattre, N., Gueguen, N., Urhausen, A., & Theisen, D. (2016). Injury risk in runners using standard or motion control shoes: A randomised controlled trial with participant and assessor blinding. *British Journal of Sports Medicine*, *50*(8), 481–487. https://doi.org/10.1136/bjsports-2015-095031

Malisoux, L., Delattre, N., Urhausen, A., & Theisen, D. (2020). Shoe cushioning influences the running injury risk according to body mass: A randomized controlled trial involving 848 recreational runners. *The American Journal of Sports Medicine*, *48*(2), 473–480.

Malisoux, L., Gette, P., Backes, A., Delattre, N., Cabri, J., & Theisen, D. (2021). Relevance of Frequency-Domain Analyses to Relate Shoe Cushioning, Ground Impact Forces and Running Injury Risk: A Secondary Analysis of a Randomized Trial With 800+ Recreational Runners. *Frontiers in Sports and Active Living*, *3*, 744658. cmedm. https://doi.org/10.3389/fspor.2021.744658

Malisoux, L., Gette, P., Delattre, N., Urhausen, A., & Theisen, D. (2022). Spatiotemporal and Ground-Reaction Force Characteristics as Risk Factors for Running-Related Injury: A Secondary Analysis of a Randomized Trial Including 800+ Recreational Runners. *The American Journal of Sports Medicine*, *50*(2), 537–544. https://doi.org/10.1177/03635465211063909

Malisoux, L., Nielsen, R. O., Urhausen, A., & Theisen, D. (2015). A step towards understanding the mechanisms of running-related injuries. *Journal of Science and Medicine in Sport*, *18*(5), 523–528. https://doi.org/10.1016/j.jsams.2014.07.014

Malisoux, L., Ramesh, J., Mann, R., Seil, R., Urhausen, A., & Theisen, D. (2015). Can parallel use of different shoes decrease running-related injury risk? *Scandanavian Journal of Medicine and Science in Sport*, *25*(1), 110–115.

Mann, R., Malisoux, L., Nührenbörger, C., Urhausen, A., Meijer, K., & Theisen, D. (2015). Association of previous injury and speed with running style and stride‐to‐stride fluctuations. *Scandinavian Journal of Medicine & Science in Sports*, *25*(6). https://doi.org/10.1111/sms.12397

Matos, S., Clemente, F., Silva, R., & Carral, J. (2020). Variations of Workload Indices Prior to Injuries: A Study in Trail Runners. *INTERNATIONAL JOURNAL OF ENVIRONMENTAL RESEARCH AND PUBLIC HEALTH*, *17*(11). https://doi.org/10.3390/ijerph17114037

Mayne, R., Bleakley, C., & Matthews, M. (2021). Use of monitoring technology and injury incidence among recreational runners: A cross-sectional study. *BMC SPORTS SCIENCE MEDICINE AND REHABILITATION*, *13*(1). https://doi.org/10.1186/s13102-021-00347-4

McKean, K. A., Manson, N. A., & Stanish, W. D. (2006). Musculoskeletal injury in the masters runners. *Clinical Journal of Sport Medicine : Official Journal of the Canadian Academy of Sport Medicine*, *16*(2), 149–154. https://doi.org/10.1097/00042752-200603000-00011

Melgares, C. P., Fry, A. C., & Sanchez, Z. (2019). Performance Motion Analysis Unable to Predict Running-Related Injury in Collegiate Distance Runners. *Journal of Sports Medicine & Allied Health Sciences: Official Journal of the Ohio Athletic Trainers’ Association*, *5*(2), 1–7. s3h.

Messier, S. P., Edwards, D. G., Martin, D. F., Lowery, R. B., Cannon, D. W., James, M. K., Curl, W. W., Read, H. M. J., & Hunter, D. M. (1995). Etiology of iliotibial band friction syndrome in distance runners. *Medicine and Science in Sports and Exercise*, *27*(7), 951–960. https://doi.org/10.1249/00005768-199507000-00002

Mohseni, M. M., Filmalter, S. E., Taylor, W. C., Vadeboncoeur, T. F., & Thomas, C. S. (2021). Factors Associated With Half- and Full-Marathon Race-Related Injuries: A 3-Year Review. *Clinical Journal of Sport Medicine : Official Journal of the Canadian Academy of Sport Medicine*, *31*(5), e277–e286. https://doi.org/10.1097/JSM.0000000000000775

Mokwena, P. L., Schwellnus, M. P., Van Rensburg, A. J., Ramagole, D. A., Boer, P., & Jordaan, E. (2022). Chronic Disease, Allergies, and Increased Years of Running Are Risk Factors Predicting Gradual Onset Running-Related Injuries in Ultramarathon Runners-SAFER XIX Study in 29 585 Race Entrants. *Clinical Journal of Sport Medicine : Official Journal of the Canadian Academy of Sport Medicine*, *32*(4), e422–e429. https://doi.org/10.1097/JSM.0000000000000949

Moreno, V., Areces, F., Ruiz-Vicente, D., Ordovás, J. M., & Del Coso, J. (2020). Influence of the ACTN3 R577X genotype on the injury epidemiology of marathon runners. *PloS One*, *15*(1), e0227548. https://doi.org/10.1371/journal.pone.0227548

Mousavi, S. H., Hijmans, J. M., Minoonejad, H., Rajabi, R., & Zwerver, J. (2021). Factors Associated With Lower Limb Injuries in Recreational Runners: A Cross-Sectional Survey Including Mental Aspects and Sleep Quality. *Journal of Sports Science & Medicine*, *20*(2), 204–215. https://doi.org/10.52082/jssm.2021.204

Mulvad, B., Nielsen, R. O., Lind, M., & Ramskov, D. (2018). Diagnoses and time to recovery among injured recreational runners in the RUN CLEVER. *PloS ONE*, *13*(10), e0204742.

Nakaoka, G., Barboza, S. D., Verhagen, E., van Mechelen, W., & Hespanhol, L. (2021). The Association Between the Acute:Chronic Workload Ratio and Running-Related Injuries in Dutch Runners: A Prospective Cohort Study. *Sports Medicine (Auckland, N.Z.)*, *51*(11), 2437–2447. https://doi.org/10.1007/s40279-021-01483-0

Napier, C., MacLean, C. L., Maurer, J., Taunton, J. E., & Hunt, M. A. (2018). Kinetic risk factors of running-related injuries in female recreational runners. *Scandinavian Journal of Medicine & Science in Sports*, *28*(10), 2164–2172. https://doi.org/10.1111/sms.13228

Nielsen, R. O., Bertelsen, M. L., Parner, E. T., Sørensen, H., Lind, M., & Rasmussen, S. (2014). Running more than three kilometers during the first week of a running regimen may be associated with increased risk of injury in obese novice runners. *International Journal of Sports Physical Therapy*, *9*(3), 338–345. cmedm.

Nielsen, R. O., Buist, I., Parner, E. T., Nohr, E. A., Sørensen, H., Lind, M., & Rasmussen, S. (2013). Predictors of running-related injuries among 930 novice runners: A 1-year prospective follow-up study. *Orthopaedic Journal of Sports Medicine*, *1*(1), 1–7. Scopus. https://doi.org/10.1177/2325967113487316

Nielsen, R. O., Buist, I., Parner, E. T., Nohr, E. A., Sørensen, H., Lind, M., & Rasmussen, S. (2014). Foot pronation is not associated with increased injury risk in novice runners wearing a neutral shoe: A 1-year prospective cohort study. *British Journal of Sports Medicine*, *48*(6), 440–447. https://doi.org/10.1136/bjsports-2013-092202

Nielsen, R. O., Cederholm, P., Buist, I., Sørensen, H., Lind, M., & Rasmussen, S. (2013). Can GPS Be Used to Detect Deleterious Progression in Training Volume Among Runners? *Journal of Strength and Conditioning Research*, *27*(6), 1471–1478. https://doi.org/10.1519/JSC.0b013e3182711e3c

Nielsen, R. Ø., Parner, E. T., Nohr, E. A., Sørensen, H., Lind, M., & Rasmussen, S. (2014). Excessive progression in weekly running distance and risk of running-related injuries: An association which varies according to type of injury. *The Journal of Orthopaedic and Sports Physical Therapy*, *44*(10), 739–747. https://doi.org/10.2519/jospt.2014.5164

Nielsen, R. O., Rønnow, L., Rasmussen, S., & Lind, M. (2014). A prospective study on time to recovery in 254 injured novice runners. *PloS One*, *9*(6), e99877. https://doi.org/10.1371/journal.pone.0099877

Onal, S., Leefers, M., Smith, B., & Cho, S. (2019). Predicting running injury using kinematic and kinetic parameters generated by an optical motion capture system. *SN APPLIED SCIENCES*, *1*(7). https://doi.org/10.1007/s42452-019-0695-x

Paquette, M. R., Milner, C. E., & Melcher, D. A. (2017). Foot contact angle variability during a prolonged run with relation to injury history and habitual foot strike pattern. *Scandinavian Journal of Medicine & Science in Sports*, *27*(2), 217–222. https://doi.org/10.1111/sms.12647

Payne, S. S., D’Errico, J., & Williams, D. S. B. (2019). An Examination of Step Frequency and the Running Readiness Scale as Predictors of Running-Related Injury in Collegiate Cross-Country Athletes. *Journal of Sports Medicine & Allied Health Sciences: Official Journal of the Ohio Athletic Trainers’ Association*, *5*(2), 1–7. s3h.

Peng, L., Seay, A. N., Montero, C., Barnes, L. L., Vincent, K. R., Conrad, B. P., Chen, C., & Vincent, H. K. (2015). Metabolic, cardiopulmonary, and gait profiles of recently injured and noninjured runners. *PM & R : The Journal of Injury, Function, and Rehabilitation*, *7*(1), 26–33. https://doi.org/10.1016/j.pmrj.2014.06.013

Perez-Morcillo, A., Gomez-Bernal, A., Gil-Guillen, V., Alfaro-Santafe, J., Alfaro-Santafe, J., Quesada, J., Lopez-Pineda, A., Orozco-Beltran, D., & Carratala-Munuera, C. (2019). Association between the Foot Posture Index and running related injuries: A case-control study. *CLINICAL BIOMECHANICS*, *61*, 217–221. https://doi.org/10.1016/j.clinbiomech.2018.12.019

Quirino, J., Santos, T. R. T., Okai-Nóbrega, L. A., De Araújo, P. A., Carvalho, R., Ocarino, J. D. M., Souza, T. R., & Fonseca, S. T. (2021). Runners with a history of injury have greater lower limb movement regularity than runners without a history of injury. *Sports Biomechanics*, 1–13. https://doi.org/10.1080/14763141.2021.1929435

RAMSKOV, D., BARTON, C., NIELSEN, R. O., & RASMUSSEN, S. (2015). High Eccentric Hip Abduction Strength Reduces the Risk of Developing Patellofemoral Pain Among Novice Runners Initiating a Self-Structured Running Program: A 1-Year Observational Study. *Journal of Orthopaedic & Sports Physical Therapy*, *45*(3), 153–161. s3h.

Ramskov, D., Jensen, M. L., Obling, K., Nielsen, R. O., Parner, E. T., & Rasmussen, S. (2013). No association between q-angle and foot posture with running-related injuries: A 10 week prospective follow-up study. *International Journal of Sports Physical Therapy*, *8*(4), 407–415. cmedm.

Ramskov, D., Rasmussen, S., Sorensen, H., Parner, E., Lind, M., & Nielsen, R. (2018). Run Clever—No difference in risk of injury when comparing progression in running volume and running intensity in recreational runners: A randomised trial. *BMJ OPEN SPORT & EXERCISE MEDICINE*, *4*(1). https://doi.org/10.1136/bmjsem-2017-000333

Ramskov, D., Rasmussen, S., Sørensen, H., Parner, E. T., Lind, M., & Nielsen, R. (2018). Progression in Running Intensity or Running Volume and the Development of Specific Injuries in Recreational Runners: Run Clever, a Randomized Trial Using Competing Risks. *The Journal of Orthopaedic and Sports Physical Therapy*, *48*(10), 740–748. https://doi.org/10.2519/jospt.2018.8062

Ramskov, D., Rasmussen, S., Sørensen, H., Parner, E. T., Lind, M., & Nielsen, R. (2022). Interactions Between Running Volume and Running Pace and Injury Occurrence in Recreational Runners: A Secondary Analysis. *Journal of Athletic Training*, *57*(6), 557–563. https://doi.org/10.4085/1062-6050-0165.21

Rasmussen, C. H., Nielsen, R. O., Juul, M. S., & Rasmussen, S. (2013). Weekly running volume and risk of running-related injuries among marathon runners. *International Journal of Sports Physical Therapy*, *8*(2), 111–120. cmedm.

Relph, N., & Small, K. (2019). The Influence of 9 Marathons Completed in 9 Days on Injury Incidence and Selected Musculoskeletal Tests. *International Journal of Athletic Therapy & Training*, *24*(3), 115–121. s3h.

Rhim, H. C., Kim, S. J., Jeon, J. S., Nam, H. W., & Jang, K.-M. (2021). Prevalence and risk factors of running-related injuries in Korean non-elite runners: A cross-sectional survey study. *The Journal of Sports Medicine and Physical Fitness*, *61*(3), 413–419. https://doi.org/10.23736/S0022-4707.20.11223-4

Roberts, W. O. (2000). A 12-yr profile of medical injury and illness for the Twin Cities Marathon. *Medicine and Science in Sports and Exercise*, *32*(9), 1549–1555. https://doi.org/10.1097/00005768-200009000-00004

Ryan, M., Elashi, M., Newsham-West, R., & Taunton, J. (2014). Examining injury risk and pain perception in runners using minimalist footwear. *British Journal of Sports Medicine*, *48*(16), 1257–1262. https://doi.org/10.1136/bjsports-2012-092061

Sanfilippo, D., Beaudart, C., Gaillard, A., Bornheim, S., Bruyere, O., & Kaux, J. (2021). What Are the Main Risk Factors for Lower Extremity Running-Related Injuries? A Retrospective Survey Based on 3669 Respondents. *ORTHOPAEDIC JOURNAL OF SPORTS MEDICINE*, *9*(11). https://doi.org/10.1177/23259671211043444

Schache, A. G., Blanch, P. D., Rath, D. A., Wrigley, T. V., & Bennell, K. L. (2005). Are anthropometric and kinematic parameters of the lumbo-pelvic-hip complex related to running injuries? *Research in Sports Medicine (Print)*, *13*(2), 127–147. https://doi.org/10.1080/15438620590956133

Schmida, E. A., Wille, C. M., Stiffler-Joachim, M. R., Kliethermes, S. A., & Heiderscheit, B. C. (2022). Vertical Loading Rate Is Not Associated with Running Injury, Regardless of Calculation Method. *Medicine and Science in Sports and Exercise*, *54*(8), 1382–1388. https://doi.org/10.1249/MSS.0000000000002917

Schwellnus, M., & Stubbs, G. (2006). Does running shoe prescription alter the risk of developing a running injury? *INTERNATIONAL SPORTMED JOURNAL*, *7*(2), 138–153.

Slabber, K., Schwellnus, M. P., Ramagole, D., Boulter, J., Dyer, M., Jordaan, E., & Sewry, N. (2023). Risk factors associated with gradual onset running-related injuries in 5770 ultramarathon race entrants—SAFER XXXII. *The Journal of Sports Medicine and Physical Fitness*. https://doi.org/10.23736/S0022-4707.23.14787-6

Sleeswijk Visser, T. S. O., van Middelkoop, M., Fokkema, T., & de Vos, R.-J. (2021). The socio-economic impact of running-related injuries: A large prospective cohort study. *Scandinavian Journal of Medicine & Science in Sports*, *31*(10), 2002–2009. https://doi.org/10.1111/sms.14016

Small, K., & Relph, N. (2018). Musculoskeletal Injury Rates in Multiday Marathon Runners Performing Ten Consecutive Marathons on a Repeat Course. *Journal of Athletic Enhancement*, *07*(01). https://doi.org/10.4172/2324-9080.1000280

Smits, D.-W., Backx, F., Van Der Worp, H., Van Middelkoop, M., Hartgens, F., Verhagen, E., Kluitenberg, B., & Huisstede, B. (2019). Validity of injury self-reports by novice runners: Comparison with reports by sports medicine physicians. *Research in Sports Medicine*, *27*(1), 72–87. https://doi.org/10.1080/15438627.2018.1492399

Smits, D.-W., Huisstede, B., Verhagen, E., Van Der Worp, H., Kluitenberg, B., Van Middelkoop, M., Hartgens, F., & Backx, F. (2016). Short-Term Absenteeism and Health Care Utilization Due to Lower Extremity Injuries Among Novice Runners: A Prospective Cohort Study. *Clinical Journal of Sport Medicine*, *26*(6), 502–509. https://doi.org/10.1097/JSM.0000000000000287

Suda, E. Y., Watari, R., Matias, A. B., Taddei, U. T., & Sacco, I. C. N. (2022). Predictive Effect of Well-Known Risk Factors and Foot-Core Training in Lower Limb Running-Related Injuries in Recreational Runners: A Secondary Analysis of a Randomized Controlled Trial. *The American Journal of Sports Medicine*, *50*(1), 248–254. https://doi.org/10.1177/03635465211056329

Swanevelder, S., Sewry, N., Schwellnus, M., & Jordaan, E. (2022). Predictors of multiple injuries in individual distance runners: A retrospective study of 75,401 entrants in 4 annual races-SAFER XX. *Journal of Sport and Health Science*, *11*(3), 339–346. https://doi.org/10.1016/j.jshs.2021.11.002

Switlick, T., Kernozek, T. W., & Meardon, S. (2015). Differences in joint-position sense and vibratory threshold in runners with and without a history of overuse injury. *Journal of Sport Rehabilitation*, *24*(1), 6–12. https://doi.org/10.1123/jsr.2013-0089

Taddei, U. T., Matias, A. B., Duarte, M., & Sacco, I. C. N. (2020). Foot Core Training to Prevent Running-Related Injuries: A Survival Analysis of a Single-Blind, Randomized Controlled Trial. *The American Journal of Sports Medicine*, *48*(14), 3610–3619. https://doi.org/10.1177/0363546520969205

Taunton, J. E., Ryan, M. B., Clement, D. B., McKenzie, D. C., Lloyd-Smith, D. R., & Zumbo, B. D. (2002). A retrospective case-control analysis of 2002 running injuries. *British Journal of Sports Medicine*, *36*(2), 95–101. https://doi.org/10.1136/bjsm.36.2.95

Taunton, J. E., Ryan, M. B., Clement, D. B., McKenzie, D. C., Lloyd-Smith, D. R., & Zumbo, B. D. (2003). A prospective study of running injuries: The Vancouver Sun Run ‘In Training’ clinics. *British Journal of Sports Medicine*, *37*(3), 239–244.

Tenforde, A. S., Borgstrom, H. E., Outerleys, J., & Davis, I. S. (2019). Is Cadence Related to Leg Length and Load Rate? *The Journal of Orthopaedic and Sports Physical Therapy*, *49*(4), 280–283. https://doi.org/10.2519/jospt.2019.8420

Tenforde, A. S., Hayano, T., Jamison, S. T., Outerleys, J., & Davis, I. S. (2020). Tibial Acceleration Measured from Wearable Sensors Is Associated with Loading Rates in Injured Runners. *PM & R : The Journal of Injury, Function, and Rehabilitation*, *12*(7), 679–684. cmedm. https://doi.org/10.1002/pmrj.12275

Theisen, D., Malisoux, L., Genin, J., Delattre, N., Seil, R., & Urhausen, A. (2014). Influence of midsole hardness of standard cushioned shoes on running-related injury risk. *British Journal of Sports Medicine*, *48*(5), 371–376. https://doi.org/10.1136/bjsports-2013-092613

Tillander, B., Gauffin, H., Dahlström, Ö., & Timpka, T. (2018). Associations between recreational runners’ anti-inflammatory drug use, coping strategies, and time loss due to injury and illness during preparations for a marathon event. *The Journal of Sports Medicine and Physical Fitness*, *58*(12), 1839–1843. https://doi.org/10.23736/S0022-4707.18.07747-2

Toresdahl, B. G., Robinson, J. N., Kliethermes, S. A., Metzl, J. D., Dixit, S., Quijano, B., & Fontana, M. A. (2022). Increased Incidence of Injury Among Runners With COVID-19. *Sports Health*, *14*(3), 372–376. https://doi.org/10.1177/19417381211061144

Torres, F., Gomes, A., & da Silva, S. (2020). CHARACTERISTICS OF TRAINING AND ASSOCIATION WITH INJURIES IN RECREATIONAL ROAD RUNNERS. *REVISTA BRASILEIRA DE MEDICINA DO ESPORTE*, *26*(5), 410–414. https://doi.org/10.1590/1517-8692202026052020_0045

Tricco, A. C., Lillie, E., Zarin, W., O’Brien, K. K., Colquhoun, H., Levac, D., Moher, D., Peters, M. D. J., Horsley, T., Weeks, L., Hempel, S., Akl, E. A., Chang, C., McGowan, J., Stewart, L., Hartling, L., Aldcroft, A., Wilson, M. G., Garritty, C., … Straus, S. E. (2018). PRISMA Extension for Scoping Reviews (PRISMA-ScR): Checklist and Explanation. *Annals of Internal Medicine*, *169*(7), 467–473. https://doi.org/10.7326/M18-0850

Vadeboncoeur, T., Silvers, S., Taylor, W., Shapiro, S., Roth, J., Diehl, N., Mahoney, S., & Mohseni, M. (2012). Impact of a High Body Mass Index on Lower Extremity Injury in Marathon/Half-Marathon Participants. *JOURNAL OF PHYSICAL ACTIVITY & HEALTH*, *9*(1), 96–103. https://doi.org/10.1123/jpah.9.1.96

Valliant, P. M. (1981). Personality and injury in competitive runners. *Perceptual and Motor Skills*, *53*(1), 251–253. https://doi.org/10.2466/pms.1981.53.1.251

Van Der Does, H., Kemler, E., & Gouttebarge, V. (2023). Can running-related injuries be prevented through an online behavioural intervention in adult novice runners? Results of a randomised controlled trial. *BMJ Open Sport & Exercise Medicine*, *9*(2), e001522. https://doi.org/10.1136/bmjsem-2022-001522

van der Worp, M. P., de Wijer, A., van Cingel, R., Verbeek, A. L. M., Nijhuis-van der Sanden, M. W. G., & Staal, J. B. (2016). The 5- or 10-km Marikenloop Run: A Prospective Study of the Etiology of Running-Related Injuries in Women. *The Journal of Orthopaedic and Sports Physical Therapy*, *46*(6), 462–470. https://doi.org/10.2519/jospt.2016.6402

Van Ginckel, A., Thijs, Y., Hesar, N. G. Z., Mahieu, N., De Clercq, D., Roosen, P., & Witvrouw, E. (2009). Intrinsic gait-related risk factors for Achilles tendinopathy in novice runners: A prospective study. *Gait & Posture*, *29*(3), 387–391. cmedm. https://doi.org/10.1016/j.gaitpost.2008.10.058

van Iperen, L., de Jonge, J., Gevers, J., & Vos, S. (2022). Linking psychological risk profiles to running-related injuries and chronic fatigue in long-distance runners: A latent profile analysis. *PSYCHOLOGY OF SPORT AND EXERCISE*, *58*. https://doi.org/10.1016/j.psychsport.2021.102082

van Iperen, L., de Jonge, J., Gevers, J., Vos, S., & Hespanhol, L. (2022). Is self-regulation key in reducing running-related injuries and chronic fatigue? A randomized controlled trial among long-distance runners. *JOURNAL OF APPLIED SPORT PSYCHOLOGY*, *34*(5), 983–1010. https://doi.org/10.1080/10413200.2021.2015479

Van Mechelek, W., Hlobil, H., Rep, M. H. G., Strobos, W., & Kemper, H. C. G. (1994). Running Injuries and Hamstring and Quadriceps Weakness and Balance: A Case-Control Study in Male Runners. *Sports Medicine, Training and Rehabilitation*, *5*(2), 83–93. Scopus. https://doi.org/10.1080/15438629409512004

van Mechelen, W., Hlobil, H., Kemper, H. C., Voorn, W. J., & de Jongh, H. R. (1993). Prevention of running injuries by warm-up, cool-down, and stretching exercises. *The American Journal of Sports Medicine*, *21*(5), 711–719. https://doi.org/10.1177/036354659302100513

Van Middelkoop, M., Kolkman, J., Van Ochten, J., Bierma-Zeinstra, S. M. A., & Koes, B. (2008a). Prevalence and incidence of lower extremity injuries in male marathon runners. *Scandinavian Journal of Medicine & Science in Sports*, *18*(2), 140–144. https://doi.org/10.1111/j.1600-0838.2007.00683.x

van Middelkoop, M., Kolkman, J., van Ochten, J., Bierma-Zeinstra, S. M. A., & Koes, B. W. (2007). Course and predicting factors of lower-extremity injuries after running a marathon. *Clinical Journal of Sport Medicine : Official Journal of the Canadian Academy of Sport Medicine*, *17*(1), 25–30. cmedm. https://doi.org/10.1097/JSM.0b013e3180305e4d

Van Middelkoop, M., Kolkman, J., Van Ochten, J., Bierma-Zeinstra, S. M. A., & Koes, B. W. (2008b). Risk factors for lower extremity injuries among male marathon runners. *Scandinavian Journal of Medicine & Science in Sports*, *18*(6), 691–697. https://doi.org/10.1111/j.1600-0838.2007.00768.x

Van Oeveren, B. T., De Ruiter, C. J., Hoozemans, M. J. M., Beek, P. J., & Van Dieën, J. H. (2019). Inter-individual differences in stride frequencies during running obtained from wearable data. *Journal of Sports Sciences*, *37*(17), 1996–2006. https://doi.org/10.1080/02640414.2019.1614137

van Poppel, D., de Koning, J., Verhagen, A. P., & Scholten-Peeters, G. G. M. (2016). Risk factors for lower extremity injuries among half marathon and marathon runners of the Lage Landen Marathon Eindhoven 2012: A prospective cohort study in the Netherlands. *Scandinavian Journal of Medicine & Science in Sports*, *26*(2), 226–234. https://doi.org/10.1111/sms.12424

van Poppel, D., Scholten-Peeters, G. G. M., van Middelkoop, M., Koes, B. W., & Verhagen, A. P. (2018). Risk models for lower extremity injuries among short- and long distance runners: A prospective cohort study. *Musculoskeletal Science & Practice*, *36*, 48–53. https://doi.org/10.1016/j.msksp.2018.04.007

van Poppel, D., Scholten-Peeters, G. G. M., van Middelkoop, M., & Verhagen, A. P. (2014). Prevalence, incidence and course of lower extremity injuries in runners during a 12-month follow-up period. *Scandinavian Journal of Medicine & Science in Sports*, *24*(6), 943–949. https://doi.org/10.1111/sms.12110

Venable, E. N., Seynaeve, L. A., Beale, S. T., Gamez, A., Jr., Domingo, A., Rosenthal, M. D., & Rauh, M. J. (2022). Relationships between Running Biomechanics, Hip Muscle Strength, and Running-Related Injury in Female Collegiate Cross-country Runners. *International Journal of Sports Physical Therapy*, *17*(6), 1053–1062. Scopus. https://doi.org/10.26603/001c.38017

Veras, P. M., Moreira, P. F., Catharino, L. L., Filho, J. E., Fonseca, D. S., & Felício, D. C. (2020). Incidence of injuries and associated factors in treadmill runners: A prospective cohort study. *Motriz. Revista de Educacao Fisica*, *26*(3). https://doi.org/10.1590/S1980-6574202000030038

Vernillo, G., Savoldelli, A., La Torre, A., Skafidas, S., Bortolan, L., & Schena, F. (2016). Injury and Illness Rates During Ultratrail Running. *International Journal of Sports Medicine*, *37*(7), 565–569. https://doi.org/10.1055/s-0035-1569347

Viljoen, C. T., Sewry, N., Schwellnus, M. P., Janse van Rensburg, D. C., Swanevelder, S., & Jordaan, E. (2021). Independent Risk Factors Predicting Gradual Onset Injury in 2824 Trail Running Race Entrants: SAFER XVIII Study. *Wilderness & Environmental Medicine*, *32*(3), 293–301. https://doi.org/10.1016/j.wem.2021.04.002

Vitez, L., Zupet, P., Zadnik, V., & Drobnič, M. (2017). Running injuries in the participants of Ljubljana Marathon. *Zdravstveno Varstvo*, *56*(4), 196–202. Scopus. https://doi.org/10.1515/sjph-2017-0027

Vlahek, P., & Matijević, V. (2018). Lower Extremity Injuries in Novice Runners: Incidence, Types, Time Patterns, Sociodemographic and Motivational Risk Factors in a Prospective Cohort Study. *Acta Clinica Croatica*, *57*(1), 31–38. https://doi.org/10.20471/acc.2018.57.01.04

Voight, A. M., Roberts, W. O., Lunos, S., & Chow, L. S. (2011). Pre- and postmarathon training habits of nonelite runners. *Open Access Journal of Sports Medicine*, *2*, 13–18. s3h.

Walter, S. D. (1989). The Ontario Cohort Study of Running-Related Injuries. *Archives of Internal Medicine*, *149*(11), 2561. https://doi.org/10.1001/archinte.1989.00390110113025

Warne, J. P., Gruber, A. H., Cheung, R., & Bonacci, J. (2021). Training and technique choices predict self-reported running injuries: An international study. *Physical Therapy in Sport : Official Journal of the Association of Chartered Physiotherapists in Sports Medicine*, *48*, 83–90. https://doi.org/10.1016/j.ptsp.2020.12.017

Wen, D. Y., Puffer, J. C., & Schmalzried, T. P. (1997). Lower extremity alignment and risk of overuse injuries in runners. *Medicine and Science in Sports and Exercise*, *29*(10), 1291–1298. https://doi.org/10.1097/00005768-199710000-00003

Wen, D. Y., Puffer, J. C., & Schmalzried, T. P. (1998). Injuries in runners: A prospective study of alignment. *Clinical Journal of Sport Medicine : Official Journal of the Canadian Academy of Sport Medicine*, *8*(3), 187–194. https://doi.org/10.1097/00042752-199807000-00005

Wiegand, K., Mercer, J. A., Navalta, J. W., Pharr, J., Tandy, R., & Freedman Silvernail, J. (2019). Running status and history: A self-report study. *Physical Therapy in Sport : Official Journal of the Association of Chartered Physiotherapists in Sports Medicine*, *39*, 8–15. https://doi.org/10.1016/j.ptsp.2019.06.003

Willems, T. M., Ley, C., Goetghebeur, E., Theisen, D., & Malisoux, L. (2021). Motion-Control Shoes Reduce the Risk of Pronation-Related Pathologies in Recreational Runners: A Secondary Analysis of a Randomized Controlled Trial. *The Journal of Orthopaedic and Sports Physical Therapy*, *51*(3), 135–143. https://doi.org/10.2519/jospt.2021.9710

Willwacher, S., Goetze, I., Fischer, K. M., & Brüggemann, G.-P. (2016). The free moment in running and its relation to joint loading and injury risk. *Footwear Science*, *8*(1), 1–11. Scopus. https://doi.org/10.1080/19424280.2015.1119890

Winter, S. C., Gordon, S., Brice, S. M., Lindsay, D., & Barrs, S. (2020). A Multifactorial Approach to Overuse Running Injuries: A 1-Year Prospective Study. *Sports Health*, *12*(3), 296–303. cmedm. https://doi.org/10.1177/1941738119888504

Yamato, T. P., Saragiotto, B. T., & Lopes, A. D. (2015). A consensus definition of running-related injury in recreational runners: A modified Delphi approach. *The Journal of Orthopaedic and Sports Physical Therapy*, *45*(5), 375–380. https://doi.org/10.2519/jospt.2015.5741
